# Genus *Smenospongia*: Untapped Treasure of Biometabolites—Biosynthesis, Synthesis, and Bioactivities

**DOI:** 10.3390/molecules27185969

**Published:** 2022-09-14

**Authors:** Sabrin R. M. Ibrahim, Sana A. Fadil, Haifa A. Fadil, Rawan H. Hareeri, Hossam M. Abdallah, Gamal A. Mohamed

**Affiliations:** 1 Preparatory Year Program, Department of Chemistry, Batterjee Medical College, Jeddah 21442, Saudi Arabia; 2 Department of Pharmacognosy, Faculty of Pharmacy, Assiut University, Assiut 71526, Egypt; 3 Department of Natural Products and Alternative Medicine, Faculty of Pharmacy, King Abdulaziz University, Jeddah 21589, Saudi Arabia; 4 Department of Clinical and Hospital Pharmacy, Faculty of Pharmacy, Taibah University, Almadinah Almunawarah 30078, Saudi Arabia; 5 Department of Pharmacology and Toxicology, Faculty of Pharmacy, King Abdulaziz University, Jeddah 21589, Saudi Arabia; 6 Department of Pharmacognosy, Faculty of Pharmacy, Cairo University, Cairo 11562, Egypt

**Keywords:** marine sponges, *Smenospongia*, indole alkaloids, sesquiterpenoids, biosynthesis, bioactivities

## Abstract

Marine sponges continue to attract remarkable attention as one of the richest pools of bioactive metabolites in the marine environment. The genus *Smenospongia* (order Dictyoceratida, family Thorectidae) sponges can produce diverse classes of metabolites with unique and unusual chemical skeletons, including terpenoids (sesqui-, di-, and sesterterpenoids), indole alkaloids, aplysinopsins, bisspiroimidazolidinones, chromenes, γ-pyrones, phenyl alkenes, naphthoquinones, and polyketides that possessed diversified bioactivities. This review provided an overview of the reported metabolites from *Smenospongia* sponges, including their biosynthesis, synthesis, and bioactivities in the period from 1980 to June 2022. The structural characteristics and diverse bioactivities of these metabolites could attract a great deal of attention from natural-product chemists and pharmaceuticals seeking to develop these metabolites into medicine for the treatment and prevention of certain health concerns.

## 1. Introduction

Marine organisms are renowned as a prosperous pool of diverse metabolites with unparalleled structural features and prominent bioactivities that display potential advantages as lead candidates for developing new pharmaceuticals [1,2]. The rate of discovery of marine metabolites has dramatically increased in the last decades [3]. It has been estimated that, since 2010, more than 15,000 marine natural metabolites have been reported [3,4].

Marine sponges (phylum Porifera) are a substantial part of the benthic biomass and have many essential ecosystem functions such as food, shelter, or regulation of substrate settlement [5]. They are sessile invertebrates that developed an efficacious chemical system based on secondary biometabolite production for communication and defense purposes [6]. In addition, the metabolites produced by sponges and their associated microorganisms are particularly beneficial to repel their surface colonization by harmful biofouling and to fight diseases [6,7]. These metabolites displayed not only unique chemical structures but also interesting bioactivities and made sponges a potential pool of lead compounds for drug discovery [8,9,10,11,12].

The genus *Smenospongia* (order Dictyoceratida, family Thorectidae) comprises 19 species [13]. The sponges of this genus have the capacity to produce diverse classes of secondary metabolites including phenyl alkenes, indole alkaloids, terpenoids (sesqui-, di-, and sesterterpenoids), aplysinopsins, bisspiroimidazolidinones, chromenes, γ-pyrones, naphthoquinones, and polyketides. Many reported metabolites from this genus possess unique and unusual chemical skeletons, often having halogen atoms. Several studies are reported on the isolation, structural characterization, and bioevaluation of *Smenospongia* sponges’ metabolites. In this current work, the potential of *Smenospongia* sponges as a natural metabolite source was highlighted. It provides an overview of the literature records upon *Smenospongia* secondary metabolites including their structures, biosynthesis, activities, and synthesis, in addition to isolation, structural characterization, and structure–activity studies, starting from 1980 until June 2022. This review could be considered as a comprehensive reference for future research on *Smenospongia* sponges and their metabolites. Additionally, the reported data here magnified the relevance of these sponges in the field of marine metabolite production and their significance in the discovery of naturally derived biometabolites.

## 2. Methodology

The literature survey for this work was accomplished by collecting the data on the performed studies on this genus from the websites of various journals and scientific databases, including Scopus, ACS (American Chemical Society), Google Scholar, PubMed, MDPI, Thieme, Science-Direct, Wiley Online Library, Bentham, Taylor & Francis, and Springer Link.

## 3. *Smenospongia* Metabolites and Their Bioactivities

### 3.1. Indole Derivatives

Several studies reported the isolation of a variety of indole alkaloids as well as closely related brominated derivatives from this genus, which are summarized in the current work along with their characterization, isolation, and bioactivities.

**Figure 1:**
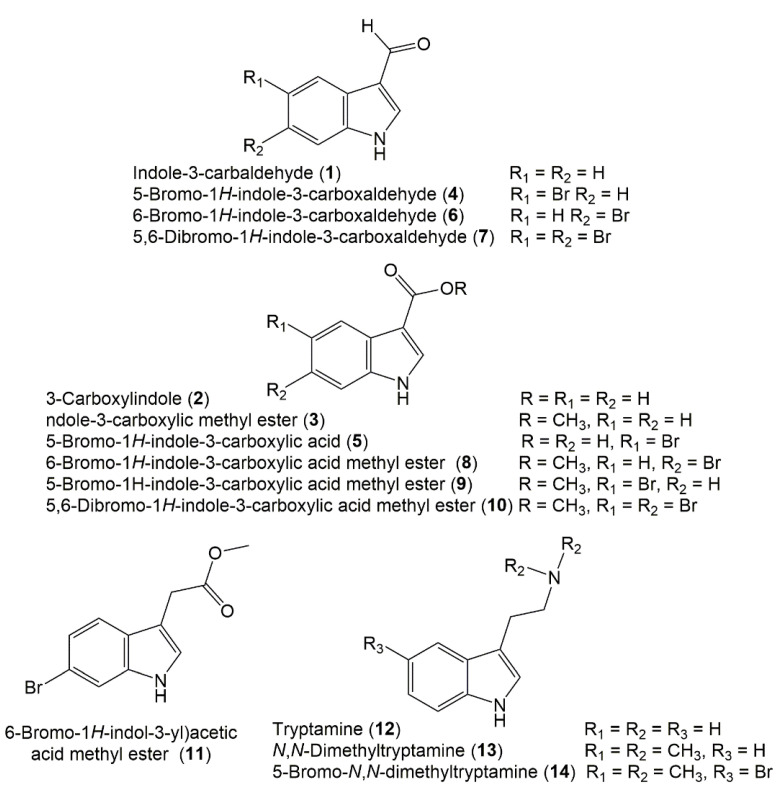
Chemical structures of indole (**1**–**14**) derivatives reported from *Smenospongia* species.

The cytotoxic activity assessment of compounds **4**–**10**, **15**, **16,** and **18**–**20** reported from Thai *Smenospongia* sp. versus MOLT-3, HepG2, A549, HuCCA-1, HeLa, HL-60, and MDA-MB-231 in the MTT assay revealed that **15** possessed cytotoxic capacity (IC_50_ 5.4 and 9.4 μM, respectively) versus MOLT-3 and HeLa cells compared with etoposide (IC_50_ 0.03 μM for MOLT-3) and doxorubicin (IC_50_ 0.38 μM for HeLa) [14]. In addition, **10** and **15** exhibited moderate activity (HeLa, IC_50_ 13.0 μM for **10** and HepG2 and HuCCA-1, IC_50_ 23.1 and 23.6 μM for **15**) [14] (Figure 1).

Djura et al. and Tymiak et al. reported the isolation of 5-bromo N,N-dimethyltryptamine (**14**) and 5,6-dibromo-N,N-dimethyltryptamine (**17**) from *S. aurea* and *S. echina* from the EtOH extract by SiO_2_ CC, prep-GC, as well as crystallization from MeOH that were characterized by NMR and chemical methods [15,16] (Supplementary Materials Table S1). Interestingly, the dibromo derivative had more antimicrobial potential versus *B. subtilis, P. atrovenetum, E. coli,* and *S. cerevisiae* than the mono-bromo analog [16] (Supplementary Materials Table S2). Furthermore, they were assessed for antidepressant potential utilizing Porsolt FST (forced swim test) and the chick anxiety-depression continuum model. Compound **17** displayed a notable antidepressant effect in the rodent FST model, whereas **14** significantly reduced the locomotor potential, revealing potential sedative influence [17].

**Figure 2:**
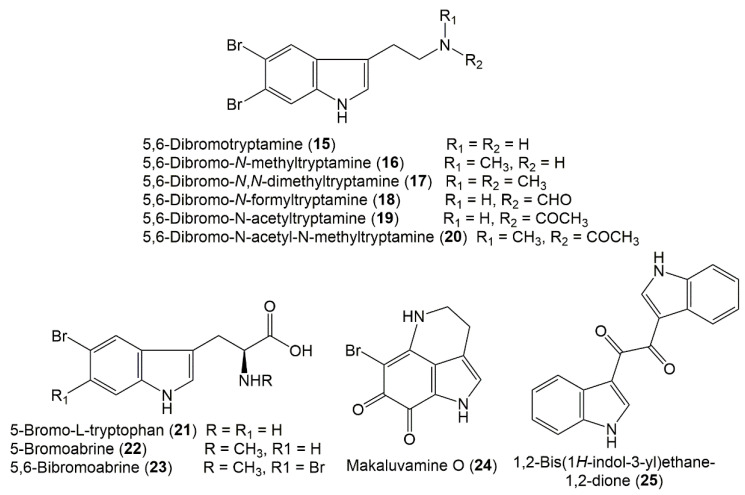
Chemical structures of indole (**15**–**25**) derivatives reported from *Smenospongia* species.

Makaluvamine O (**24**), a new pyrroloiminoquinone, along with 3-carboxylindole (**2**) and N,N-dimethyltryptamine (**13**) were obtained from *S. aurea.* Makaluvamine O (**24**) displayed moderate antimalarial potential versus *P. falciparum* (D6 Clone I) (IC_50_ 0.94 µg/mL, selectivity index > 5.1) [18]. A detailed analysis of the Philippine *Smenospongia* sp. using C-18 flash and Sephadex LH-20 column chromatography yielded four new metabolites: 5-bromo-1H-indole-3-carboxylic acid (**5**), 5-bromo-l-tryptophan (**21**), 5-bromoabrine (**22**), and 5,6-dibromoabrine (**23**), along with **15** and **24** (Figure 2).

Their cytotoxic potential was assessed versus a panel of isogenic HCT-116 cell lines consisting of p21 and p53 knockouts (p21^−/−^ and p53^−/−^) as well as the parental cell line of each (p21^+/+^ and p53^+/+^) in the MTT assay [19]. A p53 is a tumor suppressor gene that primarily acts via its downstream p21 protein mediator. The p53 and p21 alterations are the most known mutations occurring in human tumors. Only **15** possessed potential versus HCT-116 cell lines [19].

In 2002, McKay et al. purified a symmetric bisindole dimer: 1,2-Bis(1*H*-indol-3-yl)ethane-1,2-dione (**25**), in addition to **1**, **6**, and **12** from *Smenospongia* sp. MeOH extract using DIOL-bonded SiO_2_ CC (i-PrOH:hexane) and HPLC [20]. Compound **25** featured a conjugated diketo group that is related to hyrotisin B previously isolated from *Hyrtios erecta* [21]. Compounds **8** and **11** had antibacterial potential (MICs 6.25 and 50 µg/mL, respectively) versus *S. epidermidis* ATCC12228 in the microbroth dilution test [22].

### 3.2. Aplysinopsin Derivatives

Aplysinopsins are tryptophan-derived metabolites that vary in the indole moiety bromination pattern and variation in the C ring structure, including the position and number of N-methyl, oxidation state, absence and presence of the C-1′–C-8 double bond, and stereochemistry [23].

**Figure 3:**
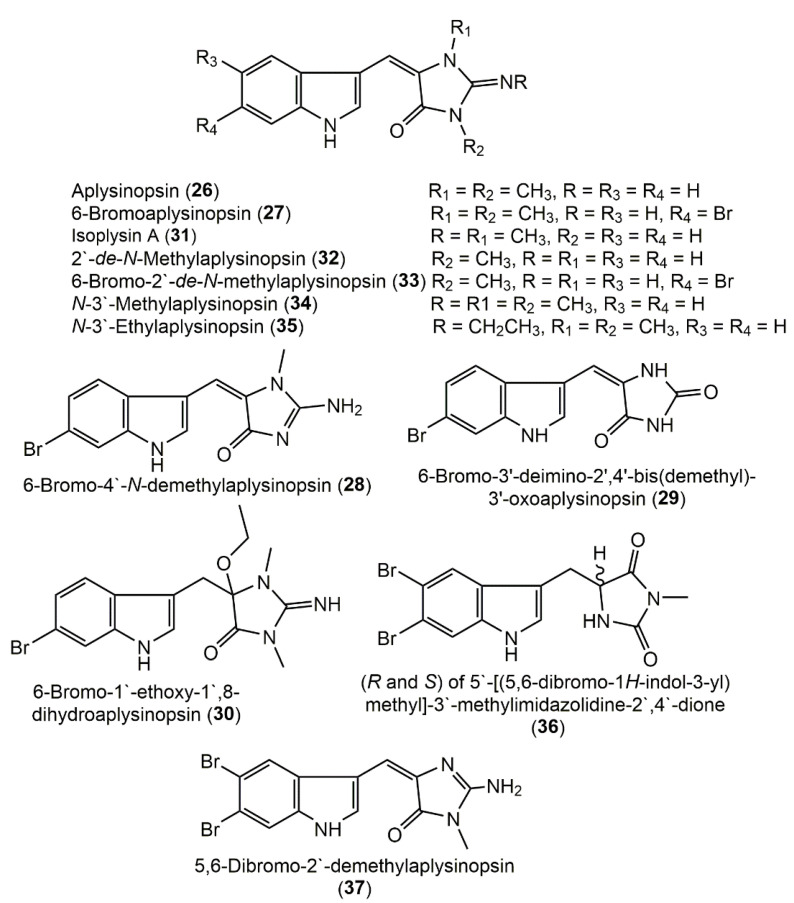
Chemical structures of aplysinopsin (**26**–**37**) derivatives reported from *Smenospongia* species.

Two new aplysinopsin derivatives, 6-bromo-aplysinopsin (**27**) and 6-bromo-4′-N-demethylaplysinopsin (**28**), were purified from the Caribbean *S. aurea* extract (toluene: MeOH 1:3) using SiO_2_ CC and crystallization from MeOH: H_2_O and elucidated using NMR, MS, and chemical synthesis (Supplementary Materials Table S3) [16]. In addition, aplysinopsins **27**–**35** separated from *S. aurea* were assessed for their in vitro antimycobacterial and antimalarial activity, as well as human 5-HT2 receptor binding antagonists in FP and FRET assays [18] (Figure 3).

5-HT_2C_ organizes food intake in mammals, whereas the 5-HT_2_A receptor displays a role in depression’s pathophysiology. Hence, these receptors’ modulators could be potential antiobesity and antidepressant agents, respectively. Only **27** exhibited antimalarial activity at endpoint 0.34 µg/mL with a selectivity index of 14, whereas **31** and **33** were moderately active (conc. 0.97 and 1.1 µg/mL, respectively, with selectivity indexes of >4.9 and >4.3, respectively). Compounds **27**, **33**, and **35** displaced high-affinity [^3^H] antagonist binding from cloned 5-HT_2C_ receptors, whereas **27** and **35** also displaced high-affinity [^3^H] antagonist binding from the 5-HT_2A_ receptor subtype, and **33** had only partial displacement at the highest concentration at the 5-HT_2A_ subtype. None of the others (**31**, **33**, and **34**) had displacement effectiveness at the highest concentration tested. Among the tested metabolites, **27** had the highest overall affinity, which was like that of endogenous serotonin at the 5-HT_2C_ receptor subtype, while **33** and **35** had a 5- to 27-fold lower affinity than serotonin. The structure–activity study revealed the role of functional groups at C-6, C-2′, and C-3′, in the binding to 5-HT_2_ human serotonin receptors. The C-3′ alkyl chain length displayed a substantial role in binding to the 5-HT receptors, and the presence of the ethyl group (as in **35**) enhanced the binding potential compared with the methyl group (as in **34**). Additionally, the Br atom at C-6 in the absence of C-3′-ethyl increased the binding affinity (as in **27** and **33** versus **32**) and selectivity to the 5-HT_2C_ receptor subtype over the 5-HT_2A_ receptor subtype. Additionally, C-2′-methylation facilitated the binding to the 5-HT_2A_ receptor subtype (as in **27** versus **33**) [20]. Chemical investigation of *Smenospongia* sp. CH_2_Cl_2_ fraction by SiO_2_ CC and HPLC yielded brominated alkaloids derivatives **26** and **30** that revealed antibacterial capacity **(**MICs 25 and 12.5 µg/mL) versus *S. epidermidis* ATCC12228 compared with vancomycin (MIC 0.625 µg/mL) in the microbroth dilution assay [22].

### 3.3. Bisspiroimidazolidinone Alkaloids

Imidazolidinones are a class of heterocyclic metabolites characterized by a nitrogen-containing five-membered ring that have received remarkable attention from medicinal chemists, as many derivatives of this scaffold class possessed variable bioactivities and are used as chiral catalysts by organic chemists [24].

**Figure 4:**
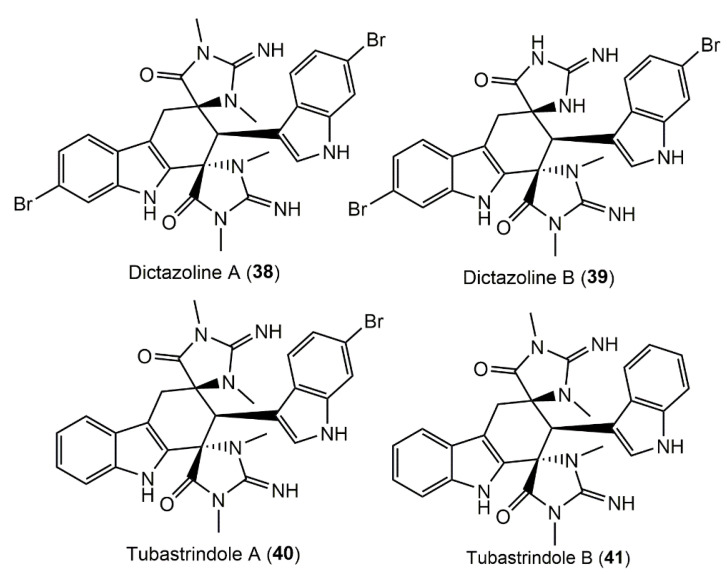
Chemical structures of bisspiroimidazolidinone (**38**–**41**) alkaloids reported from *Smenospongia* species.

Two new bisspiroimidazolidinone alkaloids, dictazolines A (**38**) and B (**39**), along with related metabolites tubastrindoles A (**40**) and B (**41**) were obtained from *S. cerebriformis* using Sephadex LH20/SiO_2_ flash column and HPLC and elucidated by NMR analyses (Supplementary Materials Table S4). Only **40** and **41** were evaluated for the inhibition of the serine and threonine kinase PKC. Initially, **41** displayed weak inhibition of PKC*δ*, whereas **40** and **41** were inactive at reducing the β-secretase-proteolytic cleavage of the amyloid precursor protein, an assay of Alzheimer’s disease treatment [25] (Figure 4).

### 3.4. Polyketides

**Figure 5:**
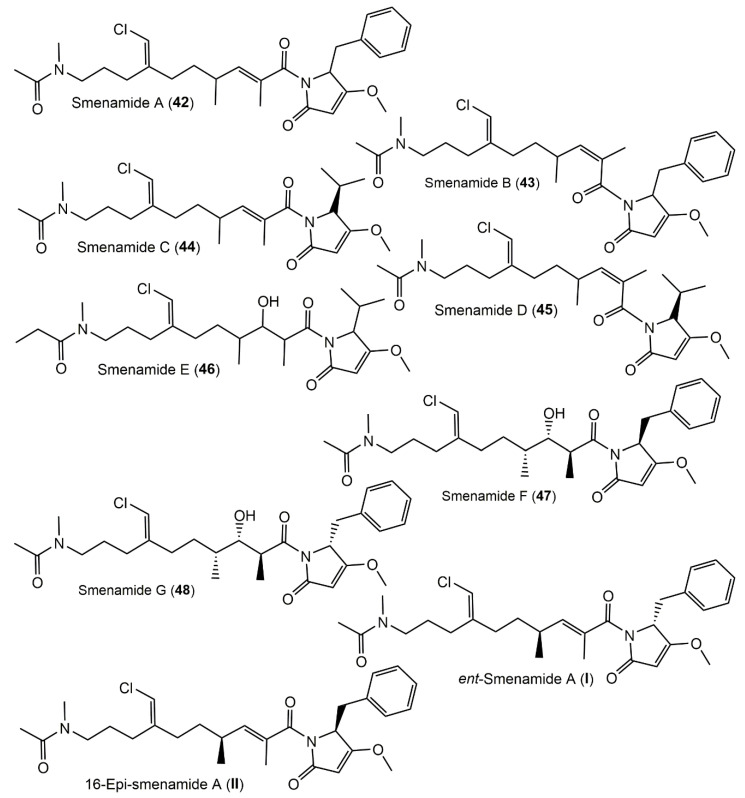
Chemical structures of smenamides (**42**–**48**) reported from *Smenospongia* species and synthetic analogs;16-epi-smenamide A and ent-smenamide.

Smenamides A (**42**) and B (**43**) are hybrid polyketide–peptide metabolites having dolapyrrolidinone, *N*-methylacetamide, and chlorovinyl moieties that were separated from Caribbean *S. aurea* and assigned using HRESIMS/MS and NMR experiments. They demonstrated no notable cytotoxic potential (conc. 30 nM) on Calu-1 in the MTT assay after 27 h of treatment; however, they (conc. 50 nM) had remarkable effectiveness (IC_50_ 48 nM for **42** and 49 nM for **43**) (Figure 5). Compound **42** exerted its powerful cytotoxic potential via a proapoptotic mechanism with increasing apoptotic cells and no or little necrotic cells, whereas in **43**, the apoptotic cells’ percentage was much less, and the necrotic cells’ percentage was high (47%, conc. 100 nM) in the Annexin-V PI/FITC kit assays. These different mechanisms could be attributed to the difference in the C13–C15 double-bond configuration.

**Figure 6:**
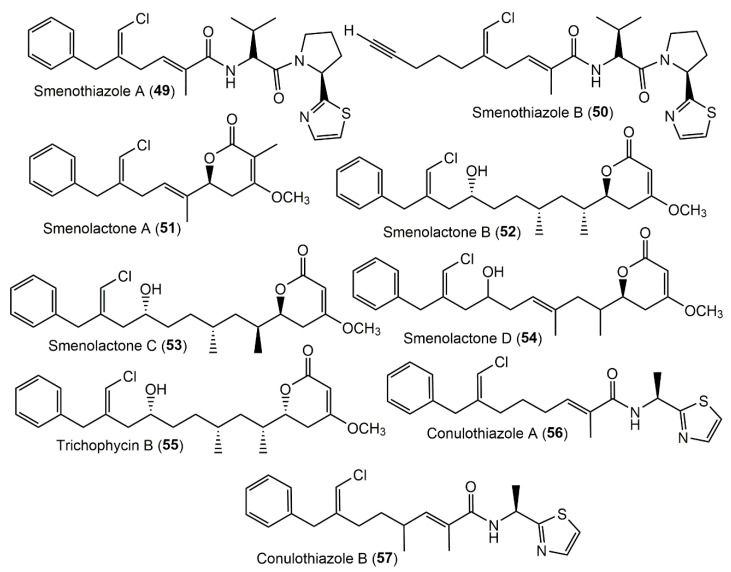
Chemical structures of smenothiazoles A (**49**) and B (**50**), smenolactones A–D (**51**–**54**), and conulothiazoles A (**56**) and B (**57**) reported from *Smenospongia* species.

These metabolites could be promising leads for designing antitumor agents [26]. Caso et al. reported the synthesis of ent-smenamide (**I**) and 16-epi-smenamide A (**II**) and established smenamide A’s 16-R configuration. The carboxylic acid moiety was created starting from *S*-citronellene via a Grignard process and two Wittig reactions. Furthermore, the Andrus protocol was used for carboxylic acid moiety coupling with either (*R*)- or (*S*)-dolapyrrolidinone [27]. In 2018, Caso et al. attempted to study if the C-16 configuration influenced the bioactivities. They synthesized 16-epi-smenamide A and eight analogs of the 16-epi-series that were tested for antiproliferative capacity versus MM (multiple myeloma) cell lines. It was found that the C-16 configuration had a slight influence on the activity since the 16-epi-analogs were active at nanomolar concentrations [28]. Interestingly, **44** and **46** revealed moderate neurotoxicity versus neuro-2A cells, whereas **45**, the geometric isomer of **44**, had no potential [29]. Using a molecular-networking dereplication strategy, two new members of smenamides, **47** and **48**, were separated through Rp-HPLC separations and elucidated by NMR, ECD, and Marfey’s analyses (Supplementary Materials Table S5). Compound **47** was a hydrated analog of **42** with 8S/13S/15S/16R/20Z-configuration [30], whereas **48** is a C-8-epimer of **47** having 8S/13S/15S/16R/20Z-configuration. Their cytotoxic potential versus MCF-7, MDA-MB-231, and MG-63 was estimated in the xCELLIgence assays. It is noteworthy that they exerted (conc. 5 μM) a moderate selective antiproliferative capacity on MDA-MB-231 and MCF-7; however, they had no effectiveness versus MG-63. It was found that the dolapyrrolinone C-8 absolute configuration did not affect the activity [30]. From the same Caribbean *S*. *aurea* using RP-18 CC and HPLC, smenothiazoles A (**49**) and B (**50**), which are hybrid peptide–polyketide compounds, were separated; these are biogenetically related and structurally vary from smenamides (Figure 6).

They were tested versus Calu-1, LC31, A2780, and MCF7 in the MTT assay. These metabolites displayed a potent cytotoxic potential at low nanomolar concentrations with selectivity versus ovarian cancer cells. They showed a comparable effect on LC31, Calu-1, and A2780 cell lines and a lower effect on MCF7 in the MTT assay, whereas in Annexin-V FITC/PI assays, smenothiazoles showed a strong apoptotic effect on A2780 (ovarian cancer cells) that is accompanied with an S phase decrease resulting in blocking the cellular cell-cycle G0G1 phase [31].

Four chlorinated polyketides, smenolactones A–D (**51**–**54**), and trichophycin B (**55**) were separated from *S. aurea* and characterized by NMR, ECD, and Mosher analyses. The cytotoxic capacity of **51** and **53**–**55** was evaluated versus MCF-7, PANC-1, and BxPC-3 tumor cell lines using the MTS assay and an xCELLigence System Real-Time Cell Analyzer (RTCA). They displayed cytotoxic activity at low- or submicromolar concentrations [32]. Smenolactones were found (conc. 250 and 500 nM and 1 and 2 µM for 48 h) to reduce the cell viability and increase the tumor cell doubling time, revealing their antiproliferative potential on MCF-7 cells. Notably, **53** (conc. 1 and 2 μM) was the most potent one (IC_50_ 918 and 652 nM, respectively); however, **51** and **54** (conc. 2 μM) caused less notable MCF-7 growth inhibition. For in vitro antiproliferative selectivity assessment, PANC-1 experienced induced or unaffected proliferation with **51** and **54** (conc. 500 nM and 1 μM, 48 h), while BXPC-3 displayed a delayed growth at 1 μM. The results demonstrated that the polyketide chain length and flexibility influence the activity as in **53**, which had no double bonds, and a longer chain was the most bioactive. Furthermore, lactone moiety C-5*R* configuration led to strong activity as in **51** versus **54** with C-5-*S* stereochemistry that had a weaker inhibition, whereas chlorovinylidene configuration had no dramatic influence on the activity [32].

Phytochemical investigation of the Caribbean *S. conulosa* led to the separation of two new chlorinated thiazole-involving metabolites, conulothiazoles A (**56**) and B (**57**), along with **42**, **43**, **49**, and **50** that were elucidated by NMR and MS analyses, and their absolute configuration was assigned by chemical degradation and Marfy’s and HRLCM analyses [33]. These metabolites possessed molecular features of several cyanobacterial metabolites, including a terminal thiazole ring as in barbamide and middle vinyl chloride as in jamaicamides [33].

### 3.5. Terpenoids

Terpenoids of variable carbon skeletons have been reported from this genus, including sesqui-, sester-, and diterpenoids (Supplementary Materials Table S6). These metabolites along with their reported bioactivities and synthesis were summarized here.

#### 3.5.1. Hydroquinone and Quinone Sesquiterpenoids

**Figure 7:**
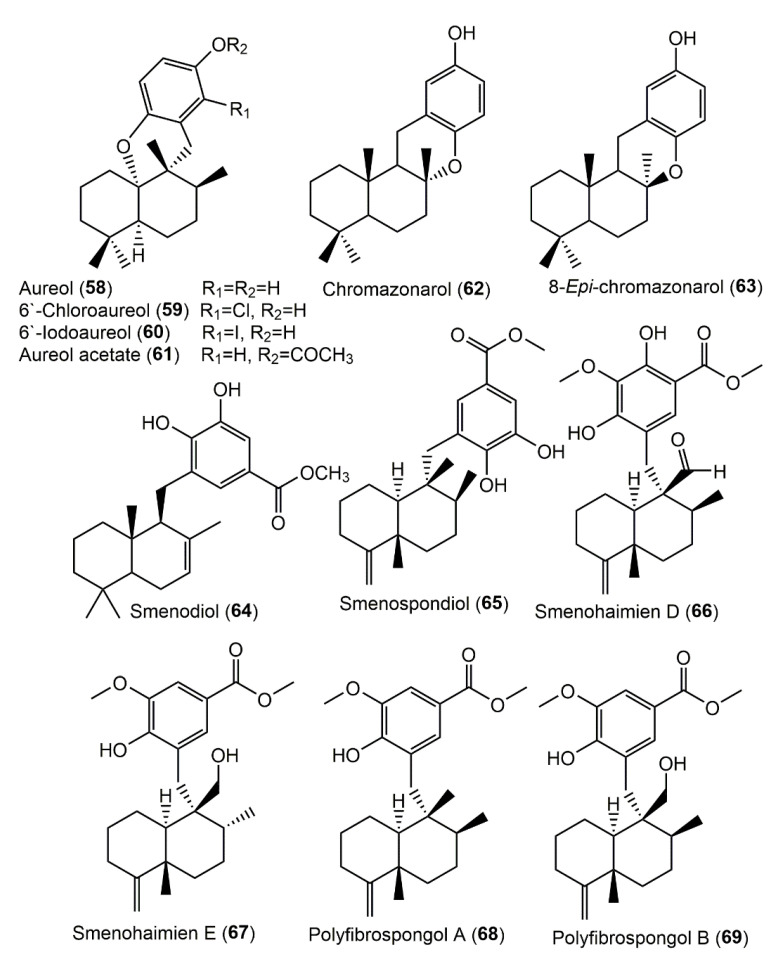
Chemical structures of hydroquinone sesquiterpenoids (**58**–**69**) reported from *Smenospongia* species.

Compounds **58** and **63** were purified from the less-polar fraction of *S. aurea* and characterized by NMR and chemical interconversion. The 5S/8S/9R/10S configuration of **58** was established based on X-ray analysis [15]. In addition, the EtOH extract of the Jamaican *S. aurea* afforded sesquiterpenes **58**, **59**, and **61** using SiO_2_ and Sephadex LH-20 (Figure 7).

**Figure 8:**
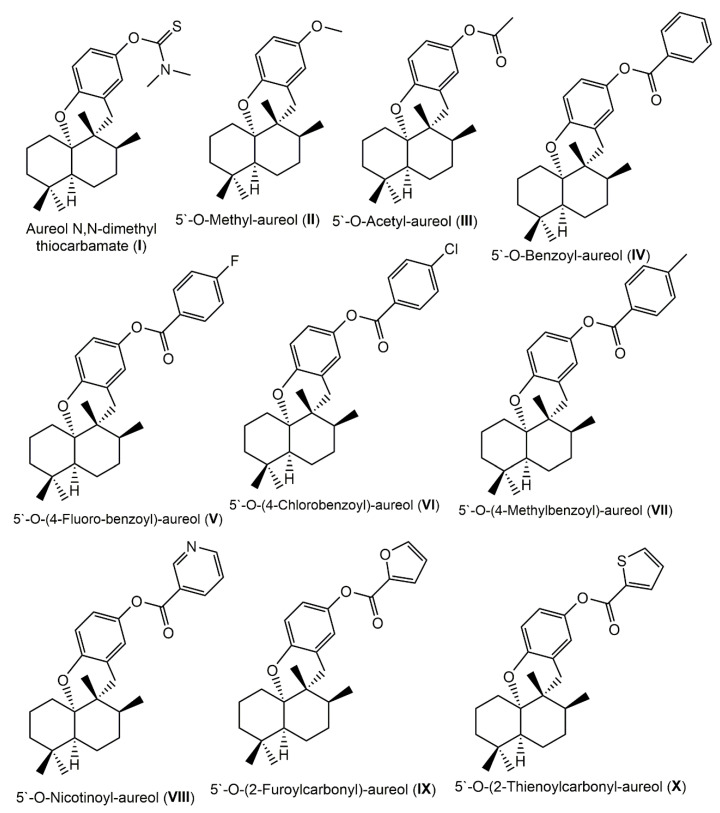
Synthetic derivatives of aureol (**I**–**X**) [20,35].

Additionally, two derivatives, aureol *N*,*N*-dimethyl thiocarbamate (**I**) and 5′-O-methyl-aureol (**II**), were semisynthesized from aureol (**58**) (Figure 8). Aureol N,N-dimethyl thiocarbamate (**I**) exhibited in vitro antimalarial potential versus the *P. falciparum* D6 clone with a selectivity index of 55, whereas it had no in vivo activity. Compounds **I** and **58** (MIC < 6.25 and >6.25 µg/mL with 100% and 31% inhibition, respectively) displayed in vitro potential versus *M. tuberculosis* H37Rv, indicating that thiocarbamate moiety highly improved the antimycobacterial activity [18]. Moreover, the EtOAc extract of *S. aurea* collected from the San Salvador Island coasts afforded **58**, **59**, and **61**. Compounds **58** and **59** were assessed for their antibacterial potential versus *B. subtilis* 6633ATCC, *B. cereus* var. *microides* 213PCI, *Staph. aureus* 6538ATCC, *S. epidermidis* 12228ATCC, *S. subflava* 7468ATCC, *S. faecalis, E. coli,* and *S. typhi* 19430ATCC. They showed antibacterial capacity with MICs ranging from 0.0008 to 0.125 for **58** and 0.5 to 0.008 mg/mL for **59** toward the tested strain [34].

Additionally, aureol (**58**) was separated by Shen et al. from the *Smenospongia* sp. nonpolar extract [36]. Its methylation yielded **II**, whereas the acylation using different acid chlorides or acetic anhydride produced **I** and **III**–**X** (Figure 8) [36]. These derivatives were evaluated in vitro for cytotoxic capacities versus VGH/Hepa59T, KB, and Hela cell lines in the MTT assay. Compound **58** had cytotoxic effectiveness (IC_50_ values of 5.77, 4.94, and 7.65 µg/mL, respectively) versus the three tested cancer cell lines compared with mitomycin (IC_50_ 0.1, 0.1, and 0.11 µg/mL, respectively). Derivatives **I**, **III**, **IX**, and **X** revealed a cytotoxic potential (IC_50_s ranging from 3.08 to 10.93 µg/mL), where **X** (IC_50_ 3.08, 3.73, and 4.15 µg/mL, respectively) was the most potent compound. The inactivity of **II** and **IV**–**VIII** indicated that 5-OHmethylation of aureol or its acylation with benzoyl, nicotinoyl, or other derivatives abolished the activity [36]. Additionally, Wang et al. reported a concise synthesis of **58** in six steps, including a BF_3_ (boron trifluoride)-catalyzed 1,2-methyl and domino 1,2-H shifts and a Ni^+2^ (nickel II)-catalyzed cross-coupling between an aryl Grignard reagent and alkyl iodide as key steps [37]. From the EtOAc-soluble fraction of *Smenospongia* sp. using SiO_2_ and HPLC, **58**–**62** were separated. Compound **60** represented the first report of iodo-sesquiterpene hydroquinone. Only **58** had moderate and weak cytotoxic (IC_50_ 14.6 and 76.4 μM, respectively) potential versus LH-60 and A549, respectively [14].

Smenospongine (**82**) is a drimane sesquiterpene-aminoquinone that was isolated as red crystals from the *Smenospongia* sp. CH_2_Cl_2_ fraction of the MeOH extract using SiO_2_ and Sephadex LH20 and characterized using NMR and CD analyses. It displayed cytotoxic potential versus L1210 (leukemia cells, LD_50_ 1.0 μg/mL) by prohibiting DNA synthesis and antibacterial capacity versus *Staph. aureus* (MIC < 5 μg/mL) and *P. aeruginosa* (strains: PYO9 and 8203S, MIC 25 μg/mL), but weak activity against *E*. *coli* (MIC 70 μg/mL) [38].

**Figure 9:**
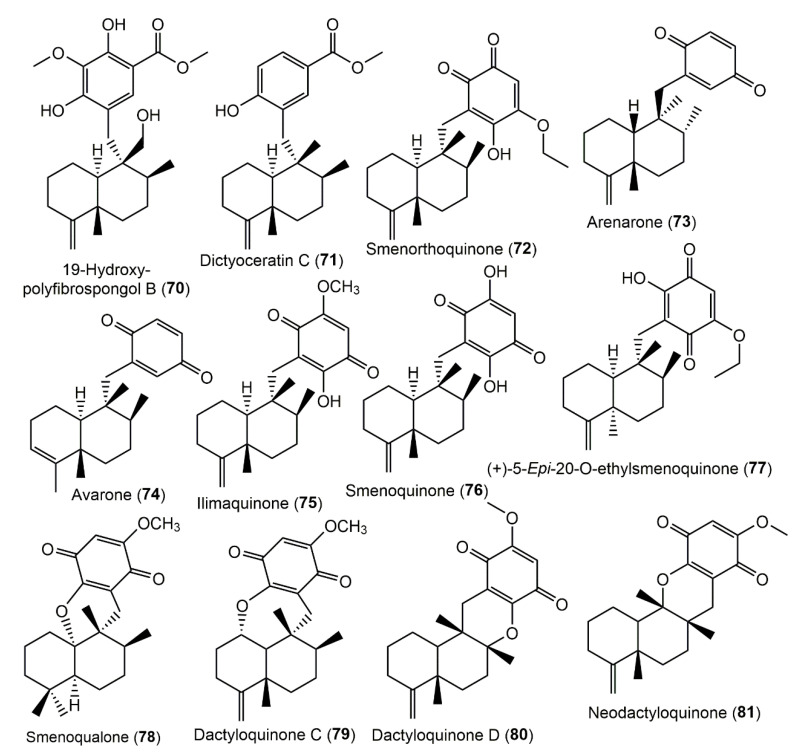
Chemical structures of hydroquinone (**70** and **71**) and quinone (**72**–**81**) sesquiterpenoids reported from *Smenospongia* species.

Various sesquiterpenoids, **65**, **72**–**76, 82**, and **84**–**86**, were separated from the *Snenospongia* sp. obtained from the Gulf of Aden, Red Sea, near Djibouti. The CH_2_Cl_2_ fraction utilizing SiO_2_/Sephadex LH-20 CC and preparative TLC was established by various spectral analyses (Figure 9).

Compounds **65, 72**, **75**, and **82** revealed a significant potential versus *Staph. aureus* (MICs 2, 10, 25, and 7 μg/mL respectively); additionally, **82** (MIC 25 μg/mL) had a potential on antibiotic-resistant *P. morganii* [38]. Furthermore, **72**, **76**, and **82** were the most powerful compounds (LD_50_ 1.5, 2.5, and 1.5 μg/mL, respectively), whereas **65**, **75**, and **85** had an LD_50_ of 4 μg/mL versus L1210 cells by assessing the inhibition of DNA synthesis estimated by ^3^H thymidine incorporation [39].

From *Snenospongia* sp., smenoqualone (**78**) was purified, which is structurally related to strongylin A separated from *Strongylophora hartmani* [40]. It had no antimicrobial and cytotoxic potential, suggesting that the free OH group on the quinone ring is substantial for the activity [41].

Do et al. reported that the **75** pretreatment remarkably boosted TRAIL-produced apoptosis in HCT-116 cells and stimulated TRAIL-caused apoptosis on colon cancer cells via increased caspase-8 and -3 activation, DNA damage, and PARP cleavage. It also lessened Bcl-xL and Bcl2 cell survival proteins, while it strongly upregulated death receptors’ DR4 and DR5 expression through the upregulation of CCAAT/CHOP (enhancer-binding protein homologous protein). The DR4, CHOP, and DR5 expression upregulation had occurred through the activation of ERK (extracellular-signal-regulated kinase) and p38 MAPK (mitogen-activated protein kinase) signaling pathways, as well as ROS generation. Therefore, **75** boosted the human colon cancer cells’ sensitivity to TRAIL-caused apoptosis via the ERK-ROS/CHOP-p38 MAPK–mediated upregulation of DR4 and DR5 expression, indicating that **75** could be developed into a chemotherapeutic agent [42].

Aerobic glycolysis is preferred more in cancer cells than the oxidative phosphorylation for ATP production. In different cancers, the upregulation of PDK1 (pyruvate dehydrogenase kinase 1) minimizes the PDH (pyruvate dehydrogenase) activity via the induction of its E1α subunit (PDHA1) phosphorylation and, subsequently, turns the energy metabolism from oxidative phosphorylation to aerobic glycolysis [43]. Therefore, PDK1 is regarded as a target for anticancer therapy. Compound **75** decreased the viability of A549, DLD-1, RKO, HEK293T, Detroit-551, and LLC cells (GI_50_ 10.5, 8.61, 50.16, 37.30, and >100 µM respectively) in the MTT sassy. It reduced the PDHA1 phosphorylation in the A549 cells by suppressing the PDK activity. It also increased oxygen consumption and decreased secretory lactate levels. Thus, it increased the oxidative phosphorylation and PDH activity while subsequently reducing cell viability via the suppression of the PDK activity. It could be a candidate for anticancer agents that acted via the PDK1 activity inhibition [44].

nAMD (neovascular age-related macular degeneration) is a common reason for irreversible vision loss in the elderly. Son et al. stated that **75** topical and oral administrations in mice and rabbits caused the inhibition and regression of laser-induced CNV (choroidal neovascularization) by β-catenin downregulation in RPE (retinal pigment epithelial) cells ((hTERT-RPE1 and ARPE-19), and it prohibited p53-mediated apoptosis induction in HUVECs (human umbilical vein endothelial cells). It repressed the expression of inflammatory and angiogenic factors and restored the E-cadherin expression in RPE cells by prohibiting the Wnt–β-catenin pathway. Therefore, it functioned through the p53–Wnt–β-catenin pathway regulation with advantages over the available cytokine-targeted anti-angiogenic therapies. It has a unique mechanism by suppressing the expression of proinflammatory and angiogenic factors and prohibiting the growth of vascular endothelial cells; it could be administered more safely, cost-effectively, and conveniently in the form of eye drops or oral drugs to patients than the currently used intravitreal drugs [45].

**Figure 10:**
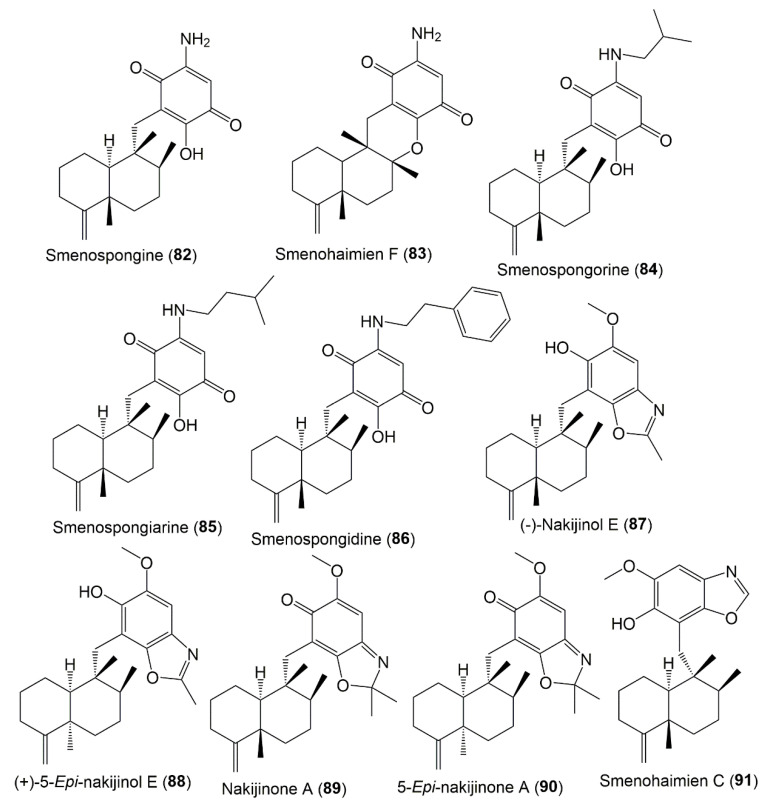
Chemical structures of amino- (**82**–**86**) and oxazole-containing (**87**–**91**) sesquiterpenoids reported from *Smenospongia* species.

From *S. aurea* and *S. cerebriformis*, **77**, **87**–**90**, and **97**–**99** were purified and assigned based on various NMR data (Figure 10). Compounds **89** and **90** have an unprecedented 2,2-dimethylbenzo[d]oxazol-6(2H)-one moiety. GIAO (gauge-invariant atomic orbital) NMR chemical shift calculations along with the application of CP3 and DP4 advanced statistics were utilized for stereochemistry determination.

**Scheme 1:**
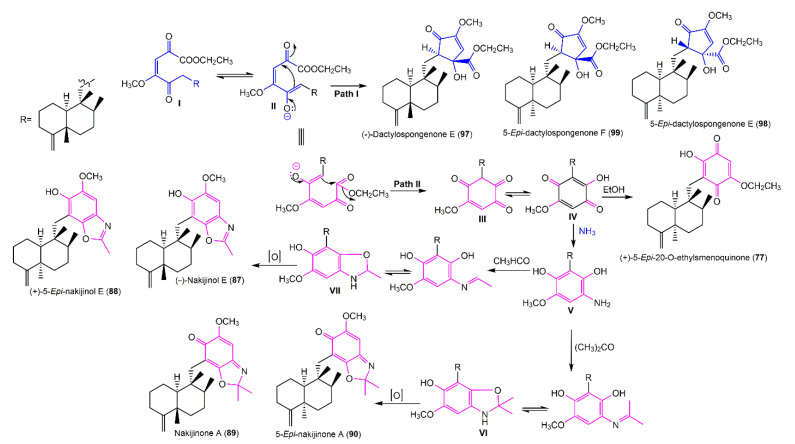
Biosynthetic pathways of **77**, **87**–**90**, and **97**–**99** [46].

The downregulation of β-catenin expression has been considered a promising approach for cytotoxic drug formulation. Compounds **77** and **88** and the mixture of **89** and **90** suppressed CRT (β-catenin response transcription) via degrading β-catenin and exhibited cytotoxic potential versus colon cancer cells through their anti-CRT potential using a CellTiter-Glo assay kit [46]. Compounds **88** and **89**/**90** mixture started to produce antiproliferative potential (conc. > 20 μM), whereas **77** began to inhibit tumor growth (conc. 1.5 and 0.75 μM) versus HCT-116 cells (IC_50_ 2.95 μM) and SW4 (IC_50_ 3.24 μM), revealing that **77** had more potent cytotoxic potential than the other compounds [46]. It was reported that a competitive intramolecular Michael addition might be involved in these metabolites’ formation. The intramolecular addition of enolate **II** onto the C-20 carbonyl would result in the generation of **97** and **99** or **98**, relying on si- or re-face addition (path **I**). Alternatively, the addition of enolate onto the C-21 carbonyl group and tautomerization followed by trans-etherification would result in **77** formation (path **II**). Additionally, reductive amination of **IV** followed by Schiff’s base formation with acetone or acetaldehyde and consecutive isomerization generates **VI** and **VII**, respectively (Scheme 1). Lastly, the oxidation of these two intermediates leads to the formation of benzoxazole moieties in **87**–**90** [46].

New sesquiterpenes, **66**, **67**, **91**, **100**, and **101**, along with **68**–**70**, **71**, and **75** were separated from *S. cerebriformis* and assigned using NMR, MS, and ECD spectra. Compounds **100** and **101** featured cyclopentenone and 4,9-friedodrim-4(11)-ene sesquiterpene skeleton with 16R/20R and 16S/20R, respectively. Compound **91** had C-15 benzoxazole moiety instead of cyclopentenone in **100** and **101**; however, **67** was structurally similar to **70** and **69** with a different C-8-configuration. Their inhibitory potential versus NO production in BV2 microglia cells stimulated with LPS in the immune-fluorescence assay using the Griess reaction was estimated [47]. Compounds **67**, **69**, **70**, **75**, **100**, and **101** (conc. 10, 20, and 40 µM) noticeably prohibited (IC_50_ ranged from 10.40 to 30.43 µM) LPS-induced NO production in BV2 cells, whereas **75** had a significant activity (IC_50_ 10.40 µM) compared with L-NMMA (IC_50_ 22.1 µM). The structure–activity study suggested that the C-14-OH group had an important role in the NO production inhibition. Thus, **75** could be a marked anti-inflammation constituent of *S. cerebriformis* [47].

Smenohaimien F (**83**), a new sesquiterpene with dialkoxy-1,4-benzoquinone moiety, in addition to **79**–**81** were reported from *S. cerebriformis* collected from Vietnam. Compound **80** showed significant cytotoxic activities on LU-1, HL-60, SK-Mel-2, HepG-2, and MCF-7 (IC_50_ ranging from 0.7 to 1.6 µg/mL) compared with ellipticine (IC_50_ 0.4 to 0.6 µg/mL), while **79** and **83** (IC_50_ ranging from 10.0 to 63.1 µg/mL) were moderately active and **81** had a weak potential in the MTT assay [48].

#### 3.5.2. Tetronic Acid and Cyclopentenone Sesquiterpenoids

**Figure 11:**
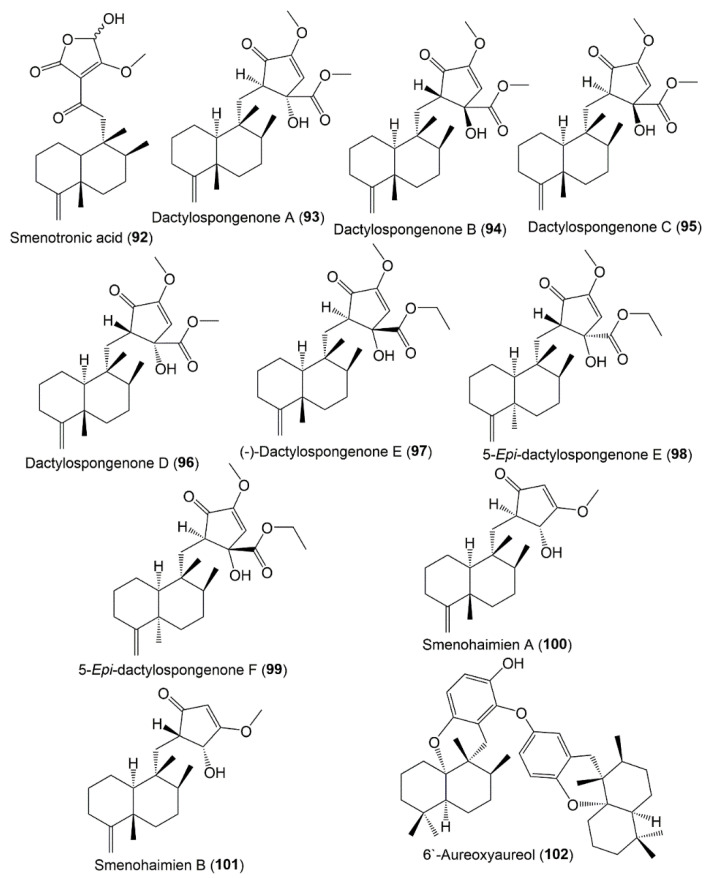
Chemical structures of tetronic acid (**92**), cyclopentenone (**93**–**101**), and dimer hydroquinone (**102**). Sesquiterpenoids reported from *Smenospongia* species.

In 1999, Bourguet-Kondracki and Guyot reported the characterization of smenotronic acid (**92**) from *Smenospongia* sp. collected from the Red Sea using NMR, IR, and UV tools [49]. It featured a rearranged drimane core with tetronic acid moiety. During storage in C_5_D_5_N, the epimerization of this compound took place to produce a mixture (1:1) of two C19 epimers that are the isomers of a dactyltronic acid, the previously reported *Dactylospongia elegans* [50]. Dactylospongenones A–D (**93**–**96**) were isolated from the *S. cerebriformis* CH_2_Cl_2_ fraction. They are sesquiterpene cyclopentenones that were formerly separated from *D. elegans* [2] (Figure 11). They were assigned by NMR analyses, and cyclopentenone’s C-16 and C-17 configuration was assigned by X-ray and CD analyses as 16R/17S, 16S/17R, 16R/17R, and 16S/17S, respectively. In the MTT assay, they revealed mild or no effectiveness against LU-1, HepG-2, HL-60, MCF-7, and SK-Mel-2 [51].

#### 3.5.3. Diterpenoids

Compounds **103** and **104** are diterpenoids reported from Vietnamese *S. cerebriformis* that had a weak cytotoxic potential versus LU-1, HL-60, SK-Mel-2, HepG-2, and MCF-7 in the MTT assay [48].

#### 3.5.4. Sesterterpenoids

**Figure 12:**
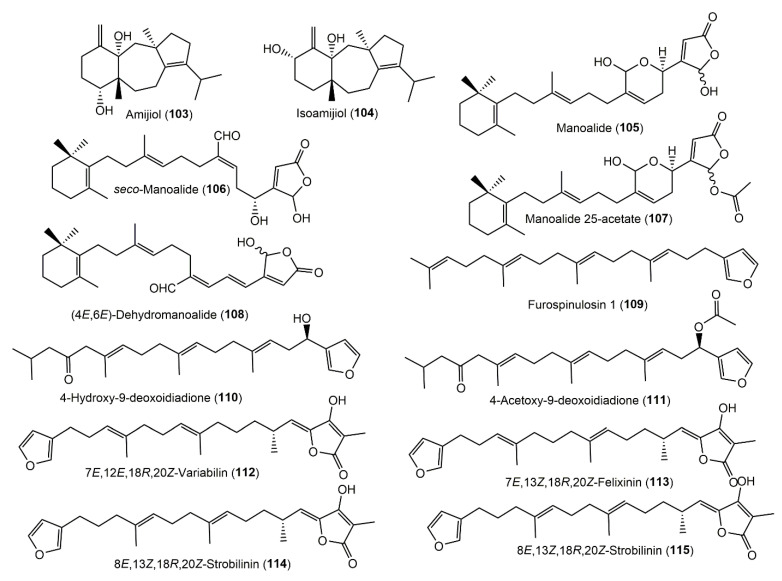
Chemical structures of diterpenoids (**103** and **104**) and sesterterpenoids (**105**–**115**) reported from *Smenospongia* species.

Four terpenoids **104**–**108** were separated using SiO_2/_Sephadex LH-20 CC and HPLC from the EtOAc extract of *Smenospongia* sp. (Figure 12). The metabolites showed an antifouling potential but no toxicity against the barnacle *Balamis Amphitrite* cypris larvae (EC_50_ 0.24, 0.80, 0.53, and 2.7 μg/mL, respectively) in the 24-well polystyrene plates assay [52]. Furthermore, two new linear furanosesterterpenes, **110** and **111**, and three new scalaranes, **116**, **117**, and **120**, were purified from *Smenospongia* sp. obtained from Gagu-Do, Korea, using RP-flash chromatography and HPLC and were characterized by NMR spectral data; **108** configuration was assigned using Mosher’s method. These metabolites revealed a cytotoxic potential (LC_50_ 31.6, 3.0, 4.9, 11.2, and 0.02 µg/mL, respectively) versus K562 (human leukemia cell line). It is noteworthy that the changes in scalarane-sesterterpenoid functional groups led to marked differences in the activity [35].

**Figure 13:**
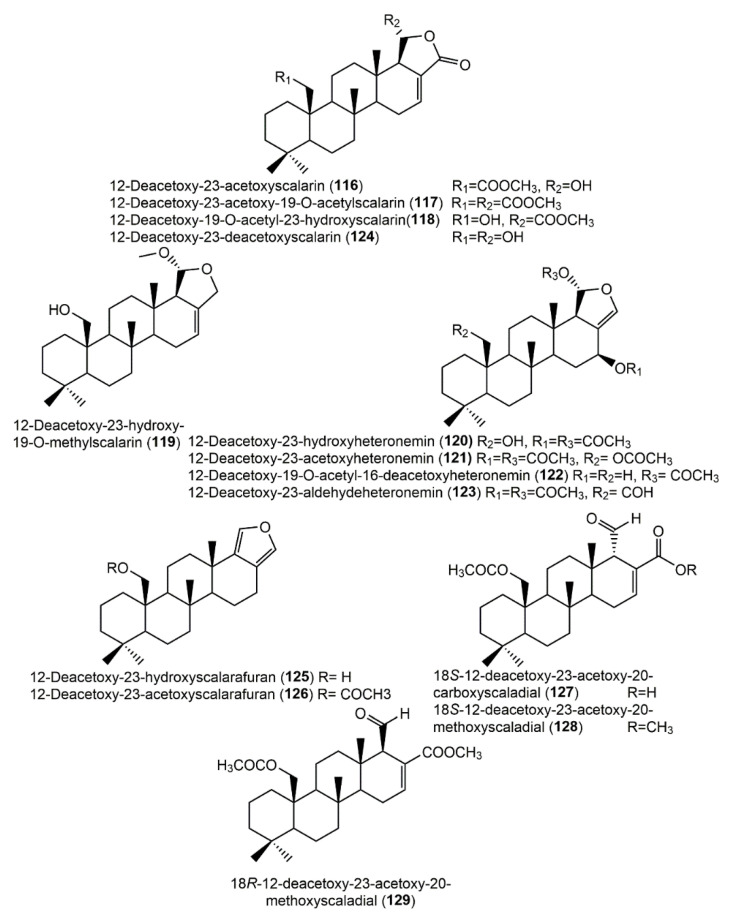
Chemical structures of scalarane sesterterpenoids (**116**–**129**) reported from *Smenospongia* species.

A new scalarane sesterterpenoid, **124**, and formerly reported **116**, **120**, **121**, **126**, and **128** were separated from the Korean *Smenospongia* sp. by reversed-phase HPLC (Figure 13).

Their activation of AMPK (5′ adenosine monophosphate-activated protein kinase) in L6 myoblast cells was tested utilizing an AMPK phosphorylation assay. Only **128** displayed a dark band that indicated the creation of phosphorylated AMPK at a concentration of 10 mM [53].

Ten new sesterterpenoids, **118**, **119**, **121**–**123**, and **125**–**129**, and known metabolites **110**, **112**–**117**, and **120** were biosynthesized by *Smenospongia* sp. obtained from Korean coastal waters and assigned by spectroscopic analyses. Compounds **123** and **127** featured unprecedented 23-aldehyde and 20-carboxylic acid, respectively, among scalarane sesterterpenoids. The isolated metabolites were evaluated for their antibacterial activity toward *Staph. aureus* (ATCC65389), *B. subtilis* (ATCC6633), *M. leuteus* (IFC12708), *P. vulgaris* (ATCC3851), *S. typhimurium* (ATCC14028), and *E. coli* (ATCC25922) in the microdilution assay, and also for the cytotoxic activity in the SRB assay against the K562 cell line and the inhibitory activity against isocitrate lyase in the colorimetric assay [54]. Compounds **112**–**115** and **129** were active versus *Staph. aureus* (MICs ranging from 25.0 to 6.25 μM), whereas **110, 112**–**115**, **117**, **118**, **120**, **122**, **125**, **126**, and **129** had a potential versus *B. subtilis (*MICs 0.78–6.25 μM) compared with ampicillin (MIC 1.56 μM). On the other side, **112**–**115**, **118**, **125**, and **129** were effective versus *M. leuteus* (MICs ranging from 12.5 to 3.12 μM), whereas **110** and **112**–**115** had activity versus *P*. *vulgaris* (MICs 3.12–6.25 μM) compared with ampicillin (MIC 3.12 μM). In the cytotoxic assay, these metabolites showed potent cytotoxic effectiveness versus the K562 cell line (LC_50_s ranging from 0.11 to 43.7 μM) compared with doxorubicin (LC_50_ 4.9 μM). Moreover, **112**–**116**, **119**, **125**, and **129** had inhibition capacity (IC_50_ ranged from 24.1 to 67.2 μM) toward microbial enzyme ICL (isocitrate lyase) compared with 3-nitropropinate (IC_50_ 6.05 μM) [54].

### 3.6. Chromene Derivatives

**Scheme 2:**
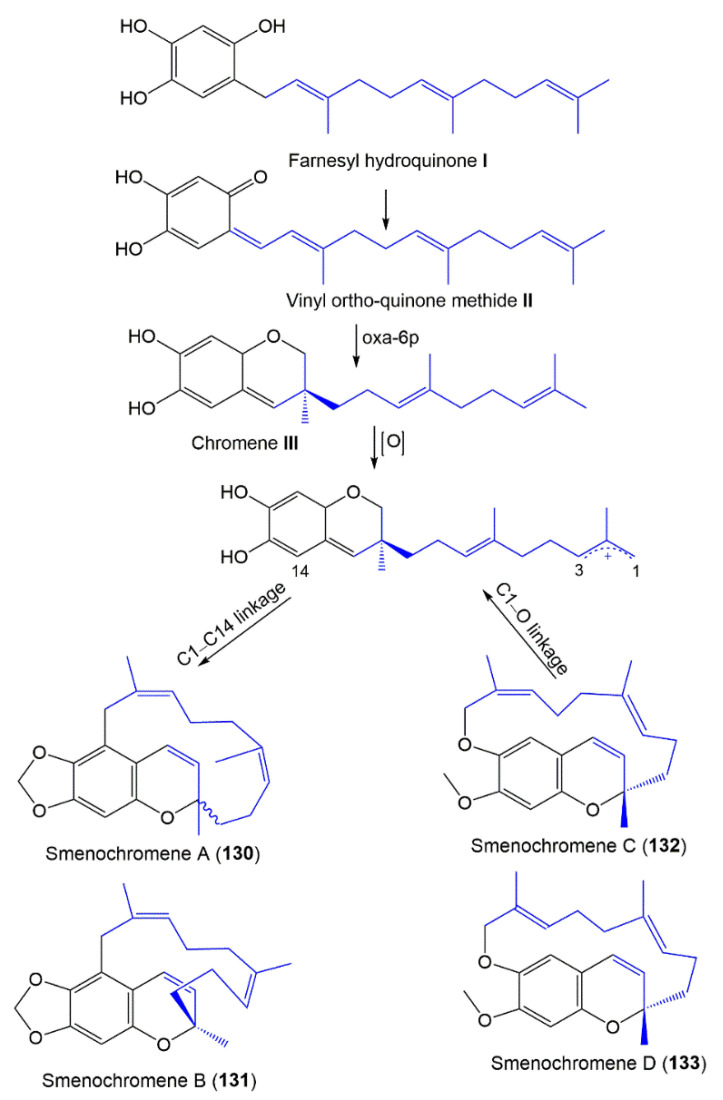
Biosynthetic pathways of smenochromenes A–D (**130**–**133**) from farnesyl hydroquinone precursor [56].

Unusual macrocyclic chromenes, smenochromenes A–D (**130**–**133**), were purified from the *Smenospongia* sp. EtOAc fraction by Sephadex LH-20 and HPLC and characterized by NMR and X-ray tools [55] (Supplementary Materials Table S7). Compound **130** was a racemate, whereas **131**–**133** were optically active. These metabolites possessed a chromene core fused to a 14-membered ring system that could be derived from farnesyl hydroquinone **I** (Scheme 2) that undergoes dehydrogenation to produce **II**. Subsequently, the oxa-6π electrocyclization of **II** affords chromene **III** featuring the hydroxy-chromene core. Furthermore, an allylic cation **IV** is formed by the terminal allylic position oxidation that undergoes various cyclizations between C1 and C14 giving the 14-membered carbocyclic **130** after that Δ^6,7^-double-bond isomerization produces **131**. On the other side, the formation of a bond between C1 and the phenolic oxygen yields **132**’s and **133′**s 16-membered heterocyclic system (Scheme 2).

**Scheme 3:**
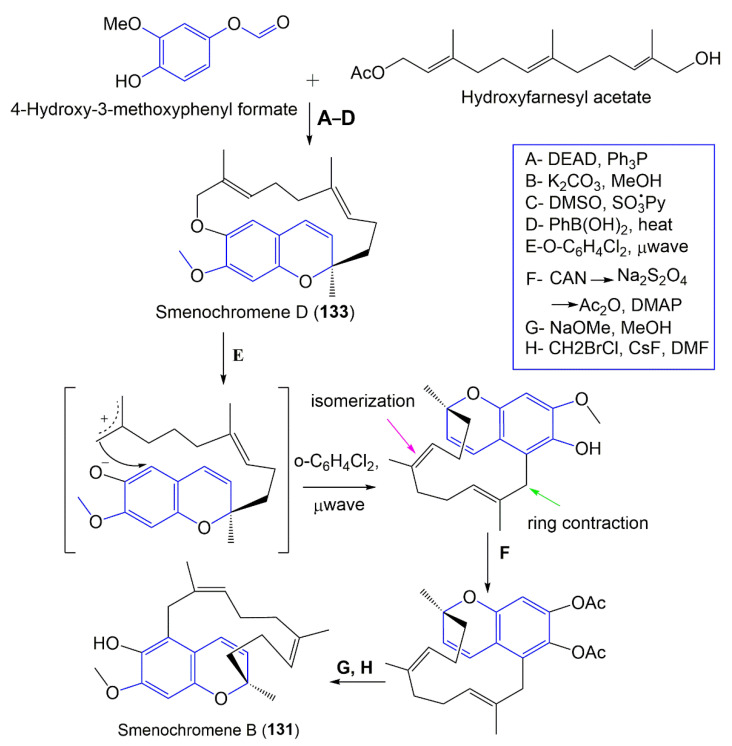
Synthesis of smenochromene B (**131**) from smenochromene D (**133**) [56].

Rosa et al. reported the synthesis of **131** via a **133** unusual rearrangement with concomitant double-bond isomerization and ring contraction (Scheme 3) [56].

### 3.7. γ-Pyrone Derivatives

The Caribbean *S. aurea* MeOH/CHCl_3_ extract afforded **134** by RP-18 CC and HPLC that featured a γ-pyrone polypropionate framework. It was assigned by NMR, MS, and ECD [57]. It is noteworthy that pyrone polypropionates are uncommon in sponges; however, they are commonly encountered in marine mollusks and bacteria [58,59].

### 3.8. Phenyl Alkenes

From Florida sponges *S. aurea* and *S. cerebriformi*, a novel phenyl alkene, **135**, with unprecedented linear phenyl alkene skeleton was separated (Supplementary Materials Table S8). Its 4R absolute configuration was established by a modified Mosher’s method. It showed in vitro cytotoxic activity versus HL-60 (IC_50_ 8.1 µM). The molecular docking study suggested that **135** produced its cytotoxic potential through the inhibition of microtubule activity [60].

### 3.9. Naphthoquinones

**Figure 14:**
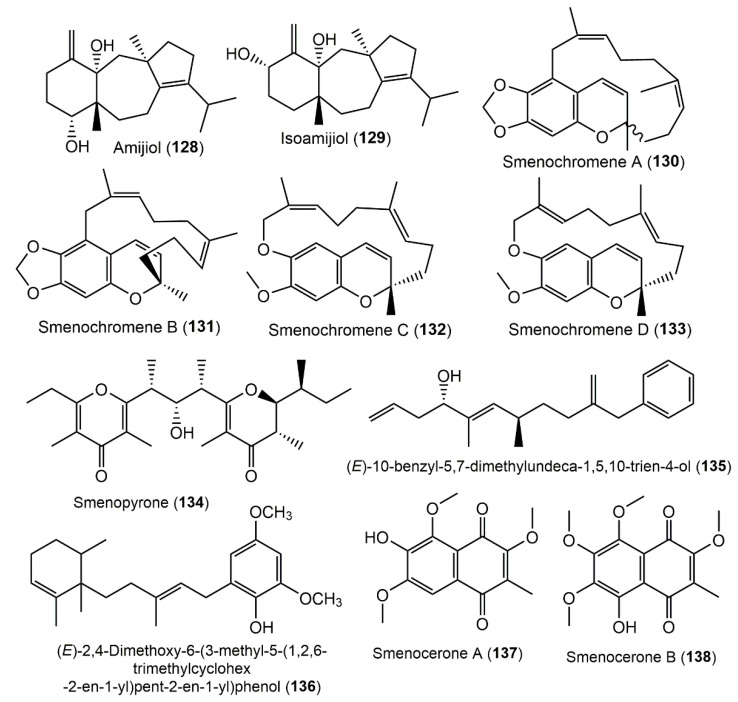
Chemical structures of chromenes (**130**–**133**), γ-pyrone (**134**), phenyl alkene (**135**), phenolic (**136**), and naphthoquinones (**137** and **138**) reported from *Smenospongia* species.

Huyen et al. purified new naphthoquinones, smenocerones A (**137**) and B (**138**), from the CH_2_Cl_2_ fraction of *S. cerebriformis* obtained from the Eastern Sea of Vietnam by SiO_2_ and RP-18 CC (Figure 14). Compound **138** (Conc. 20 µg/mL) significantly produced over 80% death of HepG-2, LU-1, MCF-7, HL-60, and SK-Mel-2 in the MTT assay with IC_50_ ranging from 3.2 to 5.7 µg/mL compared with ellipticine (IC_50_ 0.4–0.6 µg/mL) [51].

### 3.10. Fatty Acids, Sterols, and Phthalates

**Figure 15:**
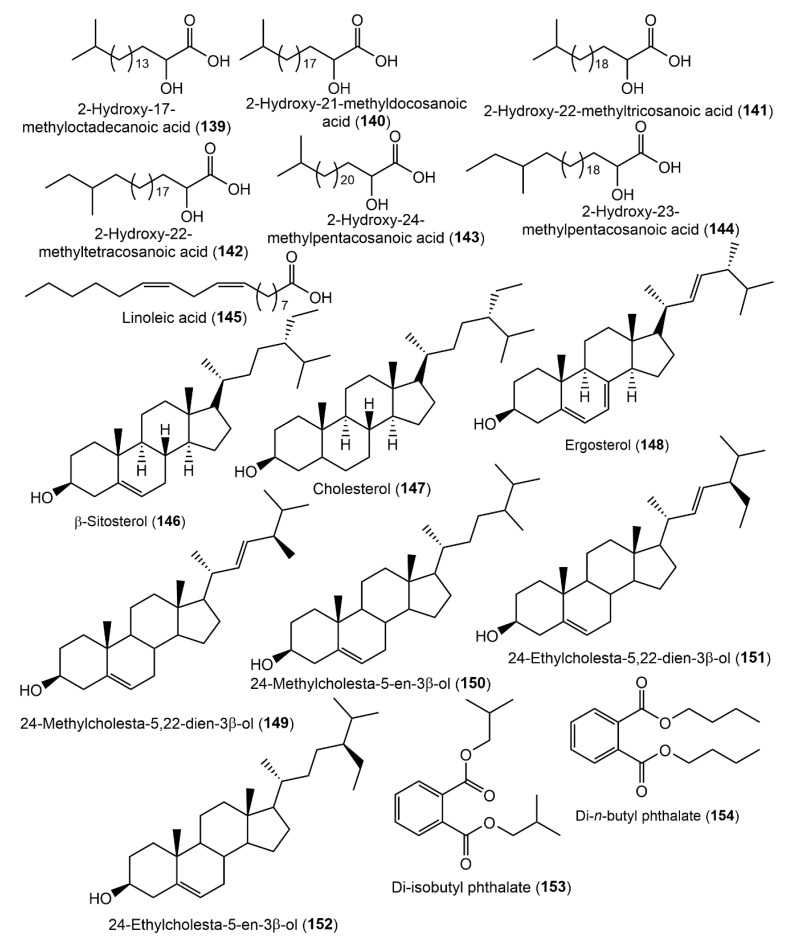
Chemical structures of fatty acids (**139**–**145**), sterols (**146**–**152**), and phthalate (**153** and **154**) reported from *Smenospongia* species.

The chemical investigation of Caribbean sponge *S. aurea* revealed the separation of six novel branched α-hydroxy fatty acids, **139**–**144**, with an anteiso or iso terminal methyl, along with a sterols mixture consisting of **147** (61%), **149** (10%), **150** (5%), **151** (2%), and **152** (22%) [61]. They were characterized by NMR, GC-MS and retention times, and chemical degradation. Additionally, from the *Smenospongia* sp. CH_2_Cl_2_–MeOH extract collected from El-Gouna–Hurghada-coasts–Red Sea–Egypt, **145**, **147**, **148**, **153**, and **154** were separated by SiO_2_ CC that were identified by NMR and GCMS analyses [62] (Figure 15, Supplementary Materials Table S9).

## 4. Biological Activity of Extracts

### 4.1. Antimicrobial Activity

*Smenospongia* sp. extracts were found to exhibit noticeable antimicrobial capacity. The CH_2_Cl_2_ extract appeared the most active versus *Staph. aureus* and *E. coli* (inhibition zone diameters (IZDs) 23 and 11 mm, respectively at conc. 500 μg/disk), whereas the MeOH extract had weaker activity (IZD 20 mm/disk versus *Staph. aureus*), and the aqueous extract displayed no activity [39].

### 4.2. Cytotoxic Activity

The CH_2_Cl_2_ extract of *Smenospongia* sp. was cytotoxic (LD_50_ 3 μg/mL) versus the L1210 cell line, whereas the MeOH and aqueous extracts were not cytotoxic toward L1210 leukemia cells until 8 μg/mL [38]; the MeOH, on the other hand, exhibited moderate cytotoxic (LC_50_ 47 µg/mL versus K562 cell line) and brine shrimp lethality (LC_50_ 160 ppm) [35].

## 5. Conclusions

**Figure 16:**
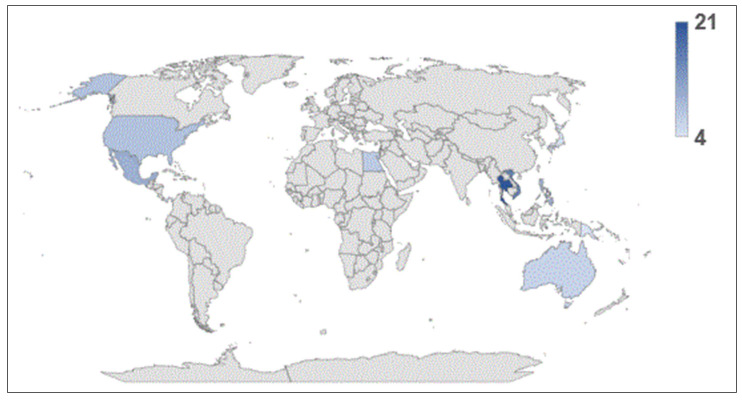
Number of reported metabolites and geographical distribution of *Smenospongia* species. The darker blue color indicated large number of metabolites reported from sponge species collected from this region; 4–21 is the range of metabolites.

Marine sponges are a wealth of biometabolites with chemical diversity that have been proven to be beneficial sources for novel drug target discovery. Among the marine sponges, *Smenospongia* sponges have been reported as a reservoir of diverse biometabolites. In the current work, *Smenospongia*, one of the most interesting sponge genera, was highlighted. The species of this genus have been collected from various regions (Figure 16). The bigger number of metabolites has been reported from the *Smenospongia* species obtained from Thailand (21 compounds), whereas the least number (4 compounds) was isolated from sponge samples that were collected from both the Milne Bay region, Papua New Guinea, and the Ninamijima and Nichinan-oshima Islands.

**Figure 17:**
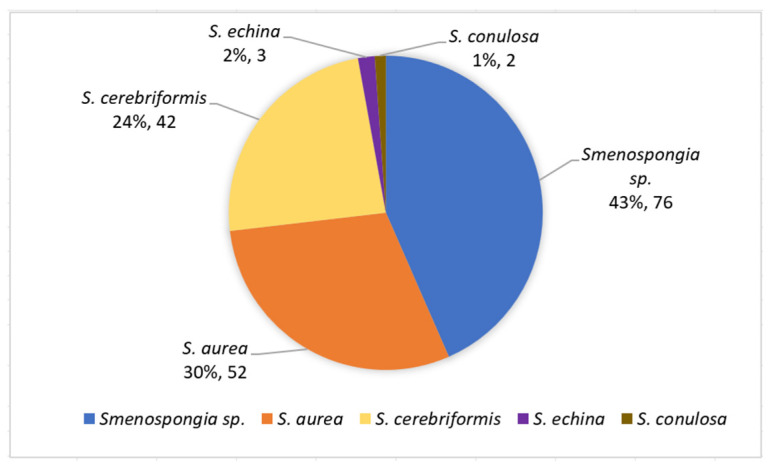
Metabolites reported from various *Smenospongia* species (% and number).

A total of 154 metabolites have been purified and characterized from various *Smenospongia* species over the last 42 years from four species: *S. aurea* (56 compounds), *S. cerebriformis* (42 compounds), *S. echina* (3 compounds), and *S. conulosa* (2 compounds), as well as unidentified *Smenospongia* species (76 compounds) (Figure 17).

**Figure 18:**
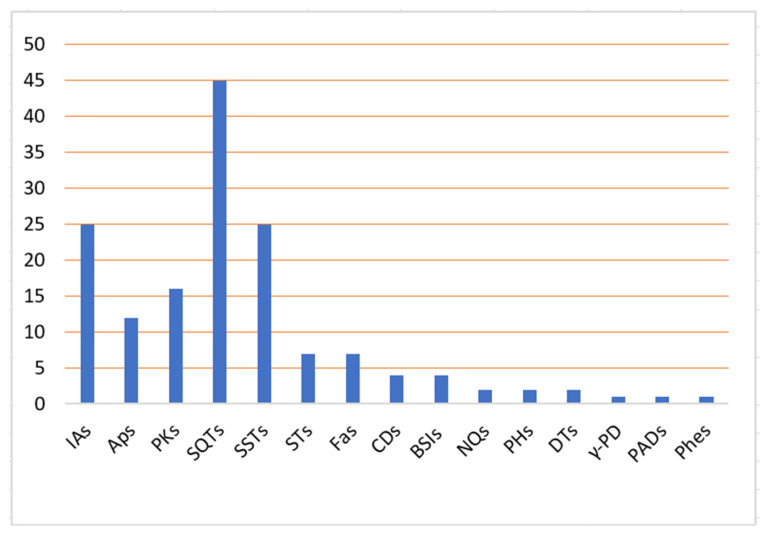
Various chemical classes of metabolites reported from various *Smenospongia* species. IAs: indole alkaloids; PKs: polyketides; APs: aplysinopsins; SQTs: sesquiterpenoids; SSTs: sesterterpenoids; STs: sterols; FAs: fatty acids; CDs: chromene derivatives; BSIs: bisspiroimidazolidinones; NQs: naphthoquinones; PHs: phthalates; DTs: diterpenoids; γ-PD: γ-pyrone derivatives; PADs: phenyl alkene derivatives; Phe: phenolics.

The results revealed that sesqui- and sesterterpenoids and indole derivatives are the major metabolites of this genus. Additionally, few studies reported the isolation of aplysinopsins, bisspiroimidazolidinones, chromenes, γ-pyrones, phenyl alkenes, naphthoquinones, polyketides, fatty acids, sterols, and phthalates (Figure 18).

**Figure 19:**
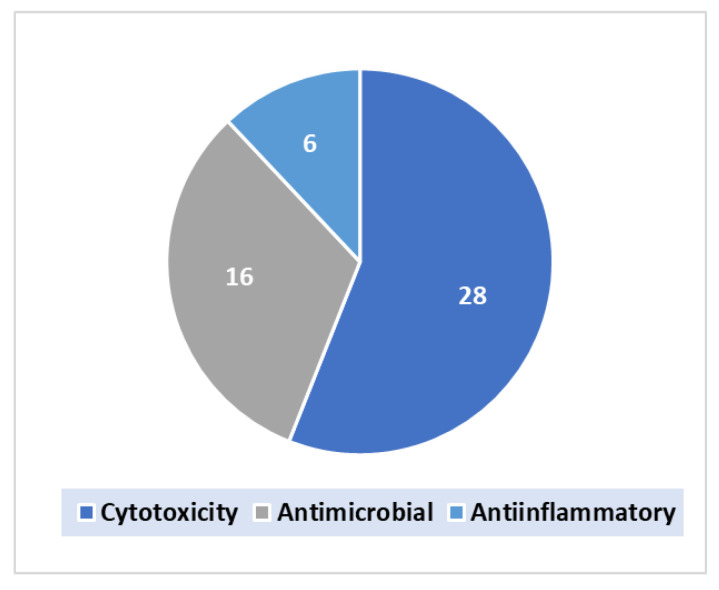
Number of compounds with potential bioactivity.

These metabolites have been assessed for diverse bioactivities, including antimalarial, antimicrobial, cytotoxic, 5-HT2 receptor antagonistic, antifouling, and anti-inflammation. Some metabolites displayed moderated to potent cytotoxic, anti-inflammation, and antimicrobial capacities (Figure 19). Among these metabolites, **80**, **117**, **119**, **120**, **126**, and **128** exhibited more potent cytotoxic capacity than standard anticancer drugs, whereas **114**, **115**, **117**, **118**, **122**, **126**, and **129** demonstrated marked antimicrobial activity versus some microbial strains.

Interestingly, *Smenospongia* genus sponges have been reported to biosynthesize chlorinated mixed polyketide–peptide compounds, including smenamides, smenothiazoles, and conulothiazoles, that shared chlorovinylidene, dolapyrrolidone ring, and terminal alkyne that are characteristics of cyanobacterial metabolites. Some of these compounds possessed potential antitumor capacity that could be promising leads for antitumor drug design. Structure–activity assessments of some reported metabolites from this genus revealed that the chemical skeletons’ nature of these metabolites and substituent patterns greatly affected the bioactivities. In addition, reported synthetic work established that the modifications of structures and replacement by some functional groups resulted in more potential and useful tags for further functionalization through click chemistry, which is a new area for drug-like molecule synthesis that can boost the drug discovery process. Unfortunately, the bioassays of some reported metabolites demonstrated no notable effectiveness, suggesting a more potential for searching and running other bioevaluations. For enriching the metabolite discovery from this genus, advanced techniques, including LC–MS–NMR, metabolomics, and UPLC–MS, could be utilized. This work aimed to be beneficial for the *Smenospongia* genus’s bioprospecting process and to bring attention to the chemical diversity of their metabolites. Finally, we think that the genus and its metabolites still warrant considerable research attention.

## Data Availability

Not applicable.

## Supplementary Materials

The following supporting information can be downloaded at: https://www.mdpi.com/article/10.3390/molecules27185969/s1, Tables S1 and S3–S9: List of reported metabolites from genus *Smenospongia* (source and place), Table S2: Biological activity of reported metabolites from genus *Smenospongia*. References [14,15,16,17,18,19,20,22,25,26,30,31,32,33,34,35,37,38,40,43,45,46,47,48,50,51,52,53,54,55,57,60,61,62,63,64] are cited in the Supplementary Materials.

## Abbreviations

A549	Human lung adenocarcinoma epithelial cell line
A2780	Human ovarian cancer cells line
AMPK	AMP-Activated protein kinase
ARPE-19	Retinal pigment epithelial cell line
ATR	Adenosine triphosphate
BV2	Microglia cells
BxPC-3	Human pancreas adenocarcinoma cell line
Calu-1	Human nonsmall-cell lung cancer cell line
CD	Circular dichroism
CH_2_Cl_2_	Dichloromethane
CHCl_3_	Chloroform
CHOP, CCAAT	Enhancer-binding protein homologous protein
CNV	Choroidal neovascularization
CRT	β-catenin response transcription
DLD-1	Human colorectal cancer cell line
DR4	Death receptor 4
DR5	Death receptor 5
DPPH	1,1-Diphenyl-2-picrylhydrazyl
EC50	Half-maximal effective concentration
ERK	Extracellular signal-regulated kinase
EtOH	Ethanol
EtOAc	Ethyl acetate
FST	Porsolt forced swim test
FRET	Fluorescence resonance energy transfer
FP	Fluorescence polarization
GCMS	Gas chromatography mass spectrometry
GIAO	Gauge-invariant atomic orbital
GI_50_	The concentration for 50% of maximal inhibition of cell
HCT-116	Human colon cancer cell line
HEK293	Human embryonic kidney cell
HepG2	Human liver cancer cell line
Hepa59T/VGH	Human liver carcinoma cell line
HeLa	Human cervical epitheloid carcinoma cell line
HL-60	Human promyelocytic leukemia cell line
HPLC	High-performance liquid chromatography
HT-29	Human colon cancer cell line
hTERT-RPE1	Retinal pigment epithelial cell lines
HuCCA-1	Human cholangiocarcinoma cell line
HUVECs	Human umbilical vein endothelial cell line
HUVSMCs	Human umbilical vein smooth muscle cells line
IC_50_	Half-maximal inhibitory concentration
ICL	Microbial enzyme isocitrate lyase
K562	Human immortalized myelogenous leukemia cell line
KB	Human oral epidermoid carcinoma cell line
L1210	Mouse lymphocytic leukemia cell line
LC31	Human lung squamous adenocarcinoma cell line
LC_50_	Lethal concentration 50
LC–MS–NMR	Liquid chromatography–mass spectrometry–nuclear magnetic resonance
LD_50_	Half maximal lethal concentration
LPS	Lipopolysaccharide
LLC	Murine Lewis lung carcinoma
L-NMMA	Nitric oxide synthase inhibitor NG-monomethyl-L-arginine
LU-1	Human lung carcinoma cell line
MCF-7	Human breast cancer cell line
MDA-MB-231	Human breast cancer cell line
MeOH	Methanol
MG-63	Human osteosarcoma cell line
MIC	Minimum inhibitory concentration
MOLT-3	Human T lymphoblast cell line
MTS	(3-(4,5-Dimethylthiazol-2-yl)-5-(3-carboxymethoxyphenyl)-2-(4-sulfophenyl)-2H-tetrazoliuminner salt)
MTT	3-(4,5-Dimethylthiazol-2-yl)-2,5-diphenyltetrazolium bromide
nAMD	Neovascular age-related macular degeneration
*n-BuOH*	n-Butanol
NMR	Nuclear magnetic resonance
NO	Nitric oxide
p38 MAPK	p38 Mitogen-activated protein kinase
P 388	Human leukemia cell line
PANC-1	Human pancreas ductal carcinoma cell line
PARP	Poly-AD Pribose polymerase
PDK1	Pyruvate dehydrogenase kinase 1
PDHA1	Phosphorylation of its E1α subunit
RKO	Human colon cancer cell line
ROS	Reactive oxygen species
RTCA	xCELLigence system real-time cell analyzer
RP-18	Reversed phase-18
SRB	Sulforhodamine B
SiO_2_ CC	Silica gel column chromatography
SK-MEL-2	Human melanoma cell line
SRB	Sulforhodamine B
SW480	Human colorectal cancer cell line
TLC	Thin layer chromatography
TRAIL	Tumor necrosis factor-related apoptosis-inducing ligand
UPLC–MS	Ultra performance liquid chromatography–tandem mass spectrometer
