# Supplementary material for: Genus Smenospongia: Untapped Treasure of Biometabolites—Biosynthesis, Synthesis, and Bioactivities
Source: Molecules. 2022 Sep 14;27(18):5969. doi: 10.3390/molecules27185969 (PMC9501515; doi:10.3390/molecules27185969)
Supplement: Supplementary file 1 [file molecules-27-05969-s001.zip › molecules-1888270-supplementary.pdf]

molecules

Supplementary Materials

Review

Genus *Smenospongia*: Untapped Treasure of Biometabolites—Biosynthesis, Synthesis, and Bioactivities

Sabrin R. M. Ibrahim [1,2,*], Sana A. Fadil [3], Haifa A. Fadil [4], Rawan H. Hareeri [5], Hossam M. Abdallah [3,6] and Gamal A. Mohamed [3]

[1] Department of Chemistry, Preparatory Year Program, Batterjee Medical College, Jeddah 21442, Saudi Arabia
[2] Department of Pharmacognosy, Faculty of Pharmacy, Assiut University, Assiut 71526, Egypt
[3] Department of Natural Products and Alternative Medicine, Faculty of Pharmacy, King Abdulaziz University, Jeddah 21589, Saudi Arabia
[4] Department of Clinical and Hospital Pharmacy, Faculty of Pharmacy, Taibah University, Almadinah Almunawarah 30078, Saudi Arabia
[5] Department of Pharmacology and Toxicology, Faculty of Pharmacy, King Abdulaziz University, Jeddah 21589, Saudi Arabia
[6] Department of Pharmacognosy, Faculty of Pharmacy, Cairo University, Cairo 11562, Egypt
* Correspondence: sabrin.ibrahim@bmc.edu.sa; Tel.: +966-581183034

Table S1. Indole derivatives reported from genus *Smenospongia*.

Compound Name	Source	Place	Mol. Wt.	Mol. Formula	Ref.
Indole-3-carbaldehyde (**1**)	*Smenospongia* sp.	South side of Porpoise Cay, Queensland, Australia	145	C_9H_7NO	[20]
	S. cerebriformis	Vinhmoc, Quangtri, Vietnam	-	-	[63]
	Smenospongia sp.	South side of Porpoise Cay, Queensland, Australia	-	-	[20]
	S. cerebriformis	Vinhmoc, Quangtri, Vietnam	-	-	[63]
3-Carboxylindole (**2**)	*S. aurea*	Discovery Bay, Jamaica	161	$C_9H_7NO_2$	[20]
Indole-3-carboxylic methyl ester (**3**)	*S. cerebriformis*	Vinhmoc, Quangtri, Vietnam	175	$C_{10}H_9NO_2$	[63]
5-Bromo-1*H*-indole-3-carboxaldehyde (**4**)	*Smenospongia* sp.	PP Island, Andaman Sea, Krabi province, Thailand	222	C_9H_6BrNO	[14]
5-Bromo-1*H*-indole-3-carboxylic acid (**5**)	*Smenospongia* sp.	Batanes, Philippines	238	$C_9H_6BrNO_2$	[19]
6-Bromo-1*H*-indole-3-carboxaldehyde (**6**)	*Smenospongia* sp.	South side of Porpoise Cay, Queensland, Australia	222	C_9H_6BrNO	[20]
	Smenospongia sp.	PP Island, Andaman Sea, Krabi province, Thailand	222	C_9H_6BrNO	[14]
5,6-Dibromo-1*H*-indole-3-carboxaldehyde (**7**)	*Smenospongia* sp.	PP Island, Andaman Sea, Krabi province, Thailand	300	$C_9H_5Br_2NO$	[14]
6-Bromo-1*H*-indole-3-carboxylic acid methyl ester (**8**)	*S. aurea*	Milne Bay region, Papua New Guinea	252	$C_{10}H_8BrNO_2$	[22]
	Smenospongia sp.	PP Island, Andaman Sea, Krabi province, Thailand	-	-	[14]
5-Bromo-1*H*-indole-3-carboxylic acid methyl ester (**9**)	*Smenospongia* sp.	PP Island, Andaman Sea, Krabi province, Thailand	252	$C_{10}H_8BrNO_2$	[14]
5,6-Dibromo-1*H*-indole-3-carboxylic acid methyl ester (**10**)	*Smenospongia* sp.	PP Island, Andaman Sea, Krabi province, Thailand	330	$C_{10}H_7Br_2NO_2$	[14]
6-Bromo-1*H*-indol-3-yl)acetic acid methyl ester (**11**)	*S. aurea*	Milne Bay region, Papua New Guinea	266	$C_{11}H_{10}BrNO_2$	[22]
Tryptamine (**12**)	*Smenospongia* sp.	South side of Porpoise Cay, Queensland, Australia	160	$C_{10}H_7N$	[20]
N,*N*-Dimethyltryptamine (**13**)	*S. aurea*	Discovery Bay, Jamaica	188	$C_{12}H_{12}N_2$	[18]
5-Bromo-*N*,*N*-dimethyltryptamine (**14**)	*S. echina*	-Puerto Morelos, Mexico -Glover and Lighthouse Reefs, Belize, Caribbean Sea	266	$C_{12}H_{15}BrN_2$	[15]
	S. aurea	-Puerto Morelos, Mexico -Glover and Lighthouse Reefs, Belize, Caribbean Sea	-	-	[15,16]
	S. aurea	San Salvador Island coasts	-	-	[34]
	S. aurea	Florida Keys, USA	-	-	[17]
5,6-Dibromotryptamine (**15**)	*Smenospongia* sp.	Batanes, Philippines	315	$C_{10}H_{10}Br_2N_2$	[19]
	Smenospongia sp.	PP Island, Andaman Sea, Krabi province, Thailand	315	$C_{10}H_{10}Br_2N_2$	[14]
5,6-Dibromo-*N*-methyltryptamine (**16**)	*Smenospongia* sp.	PP Island, Andaman Sea, Krabi province, Thailand	329	$C_{11}H_{12}Br_2N_2$	[14]
5,6-Dibromo-*N*,*N*-dimethyltryptamine (**17**)	*S. echina*	-Puerto Morelos, Mexico -Glover and Lighthouse Reefs, Belize, Caribbean Sea	343	$C_{12}H_{14}Br_2N_2$	[15,16]
	S. echina	-Puerto Morelos, Mexico -Glover and Lighthouse Reefs, Belize, Caribbean Sea	-	-	[15]

	S. aurea	Glover and Lighthouse Reefs, Belize, Caribbean Sea	-	-	[16]
	S. aurea	San Salvador Island coasts	-	-	[34]
	S. aurea	Florida Keys, USA	-	-	[17]
5,6-Dibromo-*N*- formyltryptamine (**18**)	*Smenospongia* sp.	PP Island, Andaman Sea, Krabi province, Thailand	343	$C_{11}H_{10}Br_2N_2O$	[14]
5,6-Dibromo-*N*-acetyltryptamine (**19**)	*Smenospongia* sp.	PP Island, Andaman Sea, Krabi province, Thailand	357	$C_{12}H_{12}Br_2N_2O$	[14]
5,6-Dibromo-*N*-acetyl-*N*-methyltryptamine (**20**)	*Smenospongia* sp.	PP Island, Andaman Sea, Krabi province, Thailand	371	$C_{13}H_{14}Br_2N_2O$	[14]
5-Bromo-L-tryptophan (**21**)	*Smenospongia* sp.	Batanes, Philippines	282	$C_{11}H_{11}BrN_2O_2$	[19]
5-Bromoabrine (**22**)	*Smenospongia* sp.	Batanes, Philippines	296	$C_{12}H_{13}BrN_2O_2$	[19]
5,6-Bibromoabrine (**23**)	*Smenospongia* sp.	Batanes, Philippines	373	$C_{12}H_{12}Br_2N_2O_2$	[19]
Makaluvamine O (**24**)	*S. aurea*	Discovery Bay, Jamaica	265	$C_{10}H_7BrN_2O_2$	[18]
	Smenospongia sp.	Batanes, Philippines	-	-	[19]
	S. aurea	Florida Keys, USA	-	-	[17]
1,2-Bis(1*H*-indol-3-yl)ethane-1,2-dione (**25**)	*Smenospongia* sp.	South side of Porpoise Cay, Queensland, Australia	288	$C_{18}H_{12}N_2O_2$	[20]

Table S2. Biological activity of reported metabolites from genus *Smenospongia*.

Compound Name	Biological Activity	Assay/Organism or Cell Line	Biological Results		Ref.
			Compound	Positive Control	
5,6-Dibromo-1*H*-indole-3-carboxylic acid methyl ester (**10**)	Cytotoxicity	MTT/HepG2	36.1 μM (IC_{50})	Doxorubicin 0.69 μM (IC_{50})	[14]
5,6-Dibromotryptamine (**15**)	Cytotoxicity	MTT/HCT-116 ($P^{53+/+}$)	12.6 μM (IC_{50})	Etoposide 3.4 μM (IC_{50})	[19]
		MTT/HCT-116 ($P^{53-/-}$)	53.2 μM (IC_{50})	Etoposide 17 μM (IC_{50})	[19]
		MTT/HCT-116 ($P21^{+/+}$)	85 μM (IC_{50})	Etoposide 3.4 μM (IC_{50})	[19]
		MTT/HCT-116 ($P^{21-/-}$)	63 μM (IC_{50})	Etoposide 26 μM (IC_{50})	[19]
		MTT/MOLT-3	5.4 μM (IC_{50})	Etoposide 0.03 μM (IC_{50})	[14]
		MTT/HepG2	23.1 μM (IC_{50})	Doxorubicin 0.69 μM (IC_{50})	[14]
		MTT/A549	78.6 μM (IC_{50})	Doxorubicin 0.43 μM (IC_{50})	[14]
		MTT/HuCCA-1	23.6 μM (IC_{50})	Doxorubicin 0.69 μM (IC_{50})	[14]
		MTT/HeLa	9.4 μM (IC_{50})	Doxorubicin 0.38 μM (IC_{50})	[14]
		MTT/MDA-MB-231	34.1 μM (IC_{50})	Doxorubicin 0.62 μM (IC_{50})	[14]
5,6-Dibromo-*N*-methyltryptamine (16)	Cytotoxicity	MTT/MOLT-3	46.1 μM (IC_{50})	Etoposide 0.03 μM (IC_{50})	[14]
		MTT/HepG2	23.1 μM (IC_{50})	Doxorubicin 0.69 μM (IC_{50})	[14]
		MTT/A549	78.3 μM (IC_{50})	Doxorubicin 0.43 μM (IC_{50})	[14]
		MTT/HuCCA-1	54.2 μM (IC_{50})	Doxorubicin 0.69 μM (IC_{50})	[14]
		MTT/HeLa	52.7 μM (IC_{50})	Doxorubicin 0.38 μM (IC_{50})	[14]
		MTT/HL-60	14.6 μM (IC_{50})	Etoposide 1.18 μM (IC_{50})	[14]
		MTT/MDA-MB-231	35.8 μM (IC_{50})	Doxorubicin 0.62 μM (IC_{50})	[14]
Makaluvamine O (**24**)	Cytotoxicity	MTT/HCT-116 ($P^{53+/+}$)	71 μM (IC_{50})	Etoposide 3.4 μM (IC_{50})	[19]
		MTT/HCT-116 ($P^{53-/-}$)	79 μM (IC_{50})	Etoposide 17 μM (IC_{50})	[19]
		MTT/HCT-116 ($P21^{+/+}$)	94 μM (IC_{50})	Etoposide 3.4 μM (IC_{50})	[19]
		MTT/HCT-116 ($P^{21-/-}$)	8.6 μM (IC_{50})	Etoposide 26 μM (IC_{50})	[19]
(*R* and *S*) of 5′-[(5,6-Dibromo-1*H*-indol-3-yl)methyl]-3′-methylimidazolidine-2′,4′-dione (**36**)	Cytotoxicity	MTT/MOLT-3	47.3 μM (IC_{50})	Etoposide 0.03 μM (IC_{50})	[14]
		MTT/HepG2	31.2 μM (IC_{50})	Doxorubicin 0.69 μM (IC_{50})	[14]
		MTT/A549	84.8 μM (IC_{50})	Doxorubicin 0.43 μM (IC_{50})	[14]
		MTT/HuCCA-1	87.3 μM (IC_{50})	Doxorubicin 0.69 μM (IC_{50})	[14]
		MTT/HeLa	59.9 μM (IC_{50})	Doxorubicin 0.38 μM (IC_{50})	[14]

		MTT/HeLa	13.0 μM (IC_{50})	Doxorubicin 0.38 μM (IC_{50})	[14]
5,6-Dibromo-2′-demethylaplysinopsin (**37**)	Cytotoxicity	MTT/MOLT-3	36.5 μM (IC_{50})	Etoposide 0.03 μM (IC_{50})	[14]
		MTT/HepG2	37.7 μM (IC_{50})	Doxorubicin 0.69 μM (IC_{50})	[14]
		MTT/A549	96.7 μM (IC_{50})	Doxorubicin 0.43 μM (IC_{50})	[14]
		MTT/HuCCA-1	89.2 μM (IC_{50})	Doxorubicin 0.69 μM (IC_{50})	[14]
		MTT/HeLa	75.4 μM (IC_{50})	Doxorubicin 0.38 μM (IC_{50})	[14]
		MTT/HL-60	27.0 μM (IC_{50})	Etoposide 1.18 μM (IC_{50})	[14]
		MTT/MDA-MB-231	42.7 μM (IC_{50})	Doxorubicin 0.62 μM (IC_{50})	[14]
Aureol (**58**)	Cytotoxicity	MTT/HCT-116 ($P^{53+/+}$)	15.9 μM (IC_{50})	Etoposide 3.4 μM (IC_{50})	[19]
		MTT/HCT-116 ($P^{53-/-}$)	41 μM (IC_{50})	Etoposide 17 μM (IC_{50})	[19]
		MTT/HCT-116 ($P21^{+/+}$)	73 μM (IC_{50})	Etoposide 3.4 μM (IC_{50})	[19]
		MTT/HCT-116 ($P^{21-/-}$)	61 μM (IC_{50})	Etoposide 26 μM (IC_{50})	[19]
		MTT/Hepa59T/VGH	5.77 μg/mL (IC_{50})	Mitomycin 0.1 μg/mL (IC_{50})	[36]
		MTT/KB	4.94 μg/mL (IC_{50})	Mitomycin 0.1 μg/mL (IC_{50})	[36]
		MTT/Hela	7.65 μg/mL (IC_{50})	Mitomycin 0.11 μg/mL (IC_{50})	[36]
		MTT/MOLT-3	24.8 μM (IC_{50})	Etoposide 0.03 μM (IC_{50})	[14]
		MTT/HepG2	29.2 μM (IC_{50})	Doxorubicin 0.69 μM (IC_{50})	[14]
		MTT/A549	76.4 μM (IC_{50})	Doxorubicin 0.43 μM (IC_{50})	[14]
		MTT/HuCCA-1	87.6 μM (IC_{50})	Doxorubicin 0.69 μM (IC_{50})	[14]
		MTT/HeLa	62.1 μM (IC_{50})	Doxorubicin 0.38 μM (IC_{50})	[14]
		MTT/HL-60	14.6 μM (IC_{50})	Etoposide 1.18 μM (IC_{50})	[14]
		MTT/MDA-MB-231	29.7 μM (IC_{50})	Doxorubicin 0.62 μM (IC_{50})	[14]
6′-Iodoaureol (**60**)	Cytotoxicity	MTT/MOLT-3	39.8 μM (IC_{50})	Etoposide 0.03 μM (IC_{50})	[14]
		MTT/HepG2	44.7 μM (IC_{50})	Doxorubicin 0.69 μM (IC_{50})	[14]
		MTT/A549	68.2 μM (IC_{50})	Doxorubicin 0.43 μM (IC_{50})	[14]
		MTT/HuCCA-1	63.6 μM (IC_{50})	Doxorubicin 0.69 μM (IC_{50})	[14]
		MTT/HeLa	61.4 μM (IC_{50})	Doxorubicin 0.38 μM (IC_{50})	[14]
		MTT/HL-60	43.2 μM (IC_{50})	Etoposide 1.18 μM (IC_{50})	[14]
		MTT/MDA-MB-231	44.7 μM (IC_{50})	Doxorubicin 0.62 μM (IC_{50})	[14]
Smenohaimien E (**67**)	Anti-inflammatory	NO inhibition/LPS	24.37 μM (IC_{50})	L-NMMA 22.1 μM (IC_{50})	[47]
Polyfibrospongol B (**69**)	Anti-inflammatory	NO inhibition/LPS	30.43 μM (IC_{50})	L-NMMA 22.1 μM (IC_{50})	[47]
19-Hydroxy-polyfibrospongol B (**70**)	Anti-inflammatory	NO inhibition/LPS	24.44 μM (IC_{50})	L-NMMA 22.1 μM (IC_{50})	[47]

Ilimaquinone (**75**)	Anti-inflammatory	NO inhibition/LPS	10.40 μM (IC_{50})	L-NMMA 22.1 μM (IC_{50})	[47]
Dactyloquinone C (**79**)	Cytotoxicity	MTT/LU-1	52.2 μg/mL (IC_{50})	Ellipticine 0.4 μg/mL (IC_{50})	[48]
		MTT/HL-60	51.8 μg/mL (IC_{50})	Ellipticine 0.5 μg/mL (IC_{50})	[48]
		MTT/SK-Mel-2	41.2 μg/mL (IC_{50})	Ellipticine 0.4 μg/mL (IC_{50})	[48]
		MTT/HepG-2	61.1 μg/mL (IC_{50})	Ellipticine 0.6 μg/mL (IC_{50})	[48]
		MTT/MCF-7	44.1 μg/mL (IC_{50})	Ellipticine 0.6 μg/mL (IC_{50})	[48]
Dactyloquinone D (**80**)	Cytotoxicity	MTT/LU-1	1.1 μg/mL (IC_{50})	Ellipticine 0.4 μg/mL (IC_{50})	[48]
		MTT/HL-60	0.7 μg/mL (IC_{50})	Ellipticine 0.5 μg/mL (IC_{50})	[48]
		MTT/SK-Mel-2	1.3 μg/mL (IC_{50})	Ellipticine 0.4 μg/mL (IC_{50})	[48]
		MTT/HepG-2	0.7 μg/mL (IC_{50})	Ellipticine 0.6 μg/mL (IC_{50})	[48]
		MTT/MCF-7	1.6 μg/mL (IC_{50})	Ellipticine 0.6 μg/mL (IC_{50})	[48]
Smenohaimien F (**83**)	Cytotoxicity	MTT/LU-1	10.0 μg/mL (IC_{50})	Ellipticine 0.4 μg/mL (IC_{50})	[48]
		MTT/HL-60	13.7 μg/mL (IC_{50})	Ellipticine 0.5 μg/mL (IC_{50})	[48]
		MTT/SK-Mel-2	23.5 μg/mL (IC_{50})	Ellipticine 0.4 μg/mL (IC_{50})	[48]
		MTT/HepG-2	18.1 μg/mL (IC_{50})	Ellipticine 0.6 μg/mL (IC_{50})	[48]
		MTT/MCF-7	27.9 μg/mL (IC_{50})	Ellipticine 0.6 μg/mL (IC_{50})	[48]
Smenohaimien A (**100**)	Anti-inflammatory	NO inhibition/LPS	30.13 μM (IC_{50})	L-NMMA 22.1 μM (IC_{50})	[47]
Smenohaimien B (**101**)	Anti-inflammatory	NO inhibition/LPS	28.33 μM (IC_{50})	L-NMMA 22.1 μM (IC_{50})	[47]
Furospinulosin 1 (**107**)	Cytotoxicity	MTT/HCT-116 ($P^{53+/+}$)	104 μM (IC_{50})	Etoposide 3.4 μM (IC_{50})	[19]
		MTT/HCT-116 ($P^{53-/-}$)	141 μM (IC_{50})	Etoposide 17 μM (IC_{50})	[19]
		MTT/HCT-116 ($P21^{+/+}$)	155 μM (IC_{50})	Etoposide 3.4 μM (IC_{50})	[19]
		MTT/HCT-116 ($P^{21-/-}$)	133 μM (IC_{50})	Etoposide 26 μM (IC_{50})	[19]
4-Hydroxy-9-deoxoidiadione (**108**)	Antibacterial	Microdilution/*B. subtilis*	6.25 μM (MIC)	Ampicillin 1.56 μM (MIC)	[54]
	Cytotoxicity	SRB/K562	5.7 μM (IC_{50})	Doxorubicin 4. 9 μM (IC_{50})	[54]
7*E*,12*E*,18*R*,20*Z*-Variabilin (**110**)	Antibacterial	Microdilution/*Staph. aureus*	12.5 μM (MIC)	Ampicillin 1.56 μM (MIC)	[54]
		Microdilution/*B. subtilis*	6.25 μM (MIC)	Ampicillin 1.56 μM (MIC)	[54]
		Microdilution/*M. leuteus*	6.25 μM (MIC)	Ampicillin 3.12 μM (MIC)	[54]
		Microdilution/*P. vulgaris*	6.25 μM (MIC)	Ampicillin 3.12 μM (MIC)	[54]
		Microdilution/*S. typhimurium*	6.25 μM (MIC)	Ampicillin 3.12 μM (MIC)	[54]
	Isocitrate lyase inhibition	Colorimetric/Isocitrate lyase kit	27.0 μM (IC_{50})	3-Nitropropinate 6.05 μM (IC_{50})	[54]
	Cytotoxicity	SRB/K562	43.7 μM (IC_{50})	Doxorubicin 4. 9 μM (IC_{50})	[54]

7*E*,13*Z*,18*R*,20*Z*-Felixinin (**111**)/8*E*,13*Z*,18*R*,20*Z*-Strobilinin (**112**)	Antibacterial	Microdilution/*Staph. aureus*	6.25 µM (MIC)	Ampicillin 1.56 µM (MIC)	[54]
		Microdilution/*B. subtilis*	3.12 µM (MIC)	Ampicillin 1.56 µM (MIC)	[54]
		Microdilution/*M. leuteus*	6.25 µM (MIC)	Ampicillin 3.12 µM (MIC)	[54]
		Microdilution/*P. vulgaris*	3.12 µM (MIC)	Ampicillin 3.12 µM (MIC)	[54]
		Microdilution/*S. typhimurium*	12.5 µM (MIC)	Ampicillin 3.12 µM (MIC)	[54]
	Isocitrate lyase inhibition	Colorimetric/Isocitrate Lyase kit	24.1 µM (IC_{50})	3-Nitropropinate 6.05 µM (IC_{50})	[54]
	Cytotoxicity	SRB/K562	16.9 µM (IC_{50})	Doxorubicin 4. 9 µM (IC_{50})	[54]
8*Z*,13*Z*,18*R*,20*Z*-Strobilinin (**113**)	Antibacterial	Microdilution/*Staph. aureus*	6.25 µM (MIC)	Ampicillin 1.56 µM (MIC)	[54]
		Microdilution/*B. subtilis*	6.25 µM (MIC)	Ampicillin 1.56 µM (MIC)	[54]
		Microdilution/*M. leuteus*	3.12 µM (MIC)	Ampicillin 3.12 µM (MIC)	[54]
		Microdilution/*P. vulgaris*	6.25 µM (MIC)	Ampicillin 3.12 µM (MIC)	[54]
		Microdilution/*S. typhimurium*	12.5 µM (MIC)	Ampicillin 3.12 µM (MIC)	[54]
	Isocitrate lyase inhibition	Colorimetric/Isocitrate Lyase kit	31.2 µM (IC_{50})	3-Nitropropinate 6.05 µM (IC_{50})	[54]
	Cytotoxicity	SRB/K562	3.7 µM (IC_{50})	Doxorubicin 4. 9 µM (IC_{50})	[54]
12-Deacetoxy-23-acetoxyscalarin (**114**)	Isocitrate lyase inhibition	Colorimetric/Isocitrate Lyase kit	67.2 µM (IC_{50})	3-Nitropropinate 6.05 µM (IC_{50})	[54]
	Cytotoxicity	SRB/K562	7.2 µM (IC_{50})	Doxorubicin 4. 9 µM (IC_{50})	[54]
2-Deacetoxy-23-acetoxy-19-O-acetylscalarin (**115**)	Antibacterial	Microdilution/*B. subtilis*	0.78 µM (MIC)	Ampicillin 1.56 µM (MIC)	[54]
		Microdilution/*S. typhimurium*	6.25 µM (MIC)	Ampicillin 3.12 µM (MIC)	[54]
	Cytotoxicity	SRB/K562	0.13 µM (IC_{50})	Doxorubicin 4. 9 µM (IC_{50})	[54]
12-Deacetoxy-19-O-acetyl-23-hydroxyscalarin (**116**)	Antibacterial	Microdilution/*B. subtilis*	1.56 µM (MIC)	Ampicillin 1.56 µM (MIC)	[54]
		Microdilution/*M. leuteus*	3.12 µM (MIC)	Ampicillin 3.12 µM (MIC)	[54]
		Microdilution/*S. typhimurium*	6.25 µM (MIC)	Ampicillin 3.12 µM (MIC)	[54]
	Cytotoxicity	SRB/K562	22.5 µM (IC_{50})	Doxorubicin 4. 9 µM (IC_{50})	[54]
12-Deacetoxy-23-hydroxy-19-O-methylscalarin (**117**)	Antibacterial	Microdilution/*B. subtilis*	50.0 µM (MIC)	Ampicillin 1.56 µM (MIC)	[54]
		Microdilution/*S. typhimurium*	6.25 µM (MIC)	Ampicillin 3.12 µM (MIC)	[54]
	Isocitrate lyase inhibition	Colorimetric/Isocitrate Lyase kit	42.0 µM (IC_{50})	3-Nitropropinate 6.05 µM (IC_{50})	[54]
	Cytotoxicity	SRB/K562	0.11 µM (IC_{50})	Doxorubicin 4. 9 µM (IC_{50})	[54]
12-Deacetoxy-23-hydroxyheteronemin (**118**)	Antibacterial	Microdilution/*B. subtilis*	3.12 µM (MIC)	Ampicillin 1.56 µM (MIC)	[54]

	Cytotoxicity	SRB/K562	4.9 μM (IC_{50})	Doxorubicin 4. 9 μM (IC_{50})	[54]
12-deacetoxy-23-acetoxyheteronemin (**119**)	Cytotoxicity	SRB/K562	6.8 μM (IC_{50})	Doxorubicin 4. 9 μM (IC_{50})	[54]
12-Deacetoxy-19-O-acetyl-16-deacetoxyheteronemin (**120**)	Antibacterial	Microdilution/*B. subtilis*	3.12 μM (MIC)	Ampicillin 1.56 μM (MIC)	[54]
		Microdilution/*S. typhimurium*	12.5 μM (MIC)	Ampicillin 3.12 μM (MIC)	[54]
	Cytotoxicity	SRB/K562	5.8 μM (IC_{50})	Doxorubicin 4. 9 μM (IC_{50})	[54]
12-Deacetoxy-23-aldehydeheteronemin (**121**)	Cytotoxicity	SRB/K562	17.5 μM (IC_{50})	Doxorubicin 4. 9 μM (IC_{50})	[54]
12-deacetoxy-23-acetoxyscalarafuran (**124**)	Antibacterial	Microdilution/*B. subtilis*	3.12 μM (MIC)	Ampicillin 1.56 μM (MIC)	[54]
		Microdilution/*M. leuteus*	25.0 μM (MIC)	Ampicillin 3.12 μM (MIC)	[54]
	Cytotoxicity	SRB/K562	2.3 μM (IC_{50})	Doxorubicin 4. 9 μM (IC_{50})	[54]
18*S*-12-Deacetoxy-23-acetoxy-20-carboxyscaladial (**125**)	Cytotoxicity	SRB/K562	4.2 μM (IC_{50})	Doxorubicin 4. 9 μM (IC_{50})	[54]
18*S*-12-Deacetoxy-23-acetoxy-20-methoxyscaladial (**126**)	Cytotoxicity	SRB/K562	3.7 μM (IC_{50})	Doxorubicin 4. 9 μM (IC_{50})	[54]
18*R*-12-Deacetoxy-23-acetoxy-20-methoxyscaladial (**127**)	Antibacterial	Microdilution/*Staph. aureus*	25.0 μM (MIC)	Ampicillin 1.56 μM (MIC)	[54]
		Microdilution/*B. subtilis*	0.78 μM (MIC)	Ampicillin 1.56 μM (MIC)	[54]
		Microdilution/*M. leuteus*	12.5 μM (MIC)	Ampicillin 3.12 μM (MIC)	[54]
		Microdilution/*P. vulgaris*	6.25 μM (MIC)	Ampicillin 3.12 μM (MIC)	[54]
		Microdilution/*S. typhimurium*	12.5 μM (MIC)	Ampicillin 3.12 μM (MIC)	[54]
	Isocitrate lyase inhibition	Colorimetric/Isocitrate Lyase kit	55.0 μM (IC_{50})	3-Nitropropinate 6.05 μM (IC_{50})	[54]
	Cytotoxicity	SRB/K562	8.6 μM (IC_{50})	Doxorubicin 4. 9 μM (IC_{50})	[54]
Smenocerone B (**138**)	Cytotoxicity	MTT/LU-1	5.5 μg/mL (IC_{50})	Ellipticine 0.4 μg/mL (IC_{50})	[48]
		MTT/HepG-2	3.2 μg/mL (IC_{50})	Ellipticine 0.5 μg/mL (IC_{50})	[48]
		MTT/ HL-60	4.0 μg/mL (IC_{50})	Ellipticine 0.4 μg/mL (IC_{50})	[48]
		MTT/ MCF-7	4.1 μg/mL (IC_{50})	Ellipticine 0.6 μg/mL (IC_{50})	[48]
		MTT/SK-Mel-2	5.7 μg/mL (IC_{50})	Ellipticine 0.6 μg/mL (IC_{50})	[48]

Table S3. Aplysinopsin derivatives reported from genus *Smenospongia*.

Compound Name	Source	Place	Mol. Wt.	Mol. Formula	Ref.
Aplysinopsin (**26**)	*S. aurea*	Glover and Lighthouse Reefs, Belize, Caribbean Sea	254	$C_{14}H_{14}N_4O$	[15,16]
	S. aurea	Milne Bay region, Papua New Guinea	-	-	[22]
6-Bromoaplysinopsin (**27**)	*S. aurea*	Glover and Lighthouse Reefs, Belize, Caribbean Sea	332	$C_{14}H_{13}BrN_4O$	[15,16]
	S. aurea	Discovery Bay, Jamaica	-	-	[18]
	S. aurea	Florida Keys, USA	-	-	[17]
6-Bromo-4`-*N*-demethylap-lysinopsin (**28**)	*S. aurea*	Glover and Lighthouse Reefs, Belize, Caribbean Sea	318	$C_{13}H_{11}BrN_4O$	[16]
6-Bromo-3′ -deimino-2′ ,4′ -bis(demethyl)-3′ -oxoaplysinop-sin (**29**)	*S. aurea*	San Salvador Island coasts	304	$C_{12}H_8BrN_3O_2$	[15,34]
6-Bromo-1′ -ethoxy-1′ ,8-dihy-droaplysinopsin (**30**)	*S. aurea*	Milne Bay region, Papua New Guinea	378	$C_{16}H_{19}BrN_4O_2$	[22]
Isoplysin A (**31**)	*S. aurea*	Discovery Bay, Jamaica	254	$C_{14}H_{14}N_4O$	[18]
2`-*de*-*N*-Methylaplysinopsin (**32**)	*S. aurea*	Discovery Bay, Jamaica	240	$C_{13}H_{12}N_4O$	[18]
	S. aurea	Florida Keys, USA	-	-	[17]
6-Bromo-2`-*de*-*N*-methylap-lysinopsin (**33**)	*S. aurea*	Discovery Bay, Jamaica	318	$C_{13}H_{11}BrN_4O$	[18]
	Smenospongia sp.	South side of Porpoise Cay, Queensland, Australia	-	-	[20]
N-3`-Methylaplysinopsin (**34**)	*S. aurea*	Discovery Bay, Jamaica	268	$C_{15}H_{16}N_4O$	[18]
N-3`-Ethylaplysinopsin (**35**)	*S. aurea*	Discovery Bay, Jamaica	282	$C_{16}H_{18}N_4O$	[18]
(*R* and *S*) of 5`-[(5,6-dibromo-1*H*-indol-3-yl) methyl]-3`-methylimidazolidine-2`,4`-dione (**36**)	*Smenospongia* sp.	PP Island, Andaman Sea, Krabi province, Thailand	398	$C_{13}H_{11}Br_2N_3O_2$	[14]
5,6-Dibromo-2`-demethylaplysinopsin (**37**)	*Smenospongia* sp.	PP Island, Andaman Sea, Krabi province, Thailand	395	$C_{13}H_{10}Br_2N_4O$	[14]

Table S4. Bisspiroimidazolidinone alkaloids reported from genus *Smenospongia*.

Compound Name	Source	Place	Mol. Wt.	Mol. Formula	Ref.
Dictazoline A (**38**)	*S. cerebriformis*	Hospital Point on Solarte Isle, Boca del Toro, northwest coast of Panama	664	$C_{28}H_{26}Br_2N_8O_2$	[25]
Dictazoline B (**39**)	*S. cerebriformis*	Hospital Point on Solarte Isle, Boca del Toro, northwest coast of Panama	636	$C_{26}H_{22}Br_2N_8O_2$	[25]
Tubastrindole A (**40**)	*S. cerebriformis*	Hospital Point on Solarte Isle, Boca del Toro, northwest coast of Panama	586	$C_{28}H_{27}BrN_8O_2$	[25]
Tubastrindole B (**41**)	*S. cerebriformis*	Hospital Point on Solarte Isle, Boca del Toro, northwest coast of Panama	508	$C_{28}H_{28}N_8O_2$	[25]

Table S5. Polyketides reported from genus *Smenospongia*.

Compound Name	Source	Place	Mol. Wt.	Mol. Formula	Ref.
Smenamide A (**42**)	*S. aurea*	Coast of Little Inagua, Bahamas Islands	500	$C_{27}H_{38}ClN_2O_4$	[26,30,31]
Smenamide B (**43**)	*S. aurea*	Coast of Little Inagua, Bahamas Islands	500	$C_{27}H_{38}ClN_2O_4$	[26,30,31]
Smenamide C (**44**)	*S. aurea*	Coast of Little Inagua, Bahamas Islands	452	$C_{24}H_{37}ClN_2O_4$	[30]
Smenamide D (**45**)	*S. aurea*	Coast of Little Inagua, Bahamas Islands	452	$C_{24}H_{37}ClN_2O_4$	[30]
Smenamide E (**46**)	*S. aurea*	Coast of Little Inagua, Bahamas Islands	484	$C_{25}H_{41}ClN_2O_5$	[30]
Smenamide F (**47**)	*S. aurea*	Coast of Little Inagua, Bahamas Islands	518	$C_{28}H_{39}ClN_2O_5$	[30]
Smenamide G (**48**)	*S. aurea*	Coast of Little Inagua, Bahamas Islands	518	$C_{28}H_{39}ClN_2O_5$	
Smenothiazole A (**49**)	*S. aurea*	Coast of Little Inagua, Bahamas Islands	485	$C_{26}H_{32}ClN_3O_2S$	[31]
Smenothiazole B (**50**)	*S. aurea*	Coast of Little Inagua, Bahamas Islands	461	$C_{24}H_{32}ClN_3O_2S$	[31]
Smenolactone A (**51**)	*S. aurea*	Coast of Mayaguana Island, Bahamas	346	$C_{20}H_{23}ClO_3$	[32]
Smenolactone B (**52**)	*S. aurea*	Coast of Mayaguana Island, Bahamas	420	$C_{24}H_{33}ClO_4$	[32]
Smenolactone C (**53**)	*S. aurea*	Coast of Mayaguana Island, Bahamas	420	$C_{24}H_{33}ClO_4$	[32]
Smenolactone D (**54**)	*S. aurea*	Coast of Mayaguana Island, Bahamas	418	$C_{24}H_{31}ClO_4$	[32]
Trichophycin B (**55**)	*S. aurea*	Coast of Mayaguana Island, Bahamas	420	$C_{24}H_{33}ClO_4$	[32]
Conulothiazole A (**56**)	*S. conulosa*	Coast of Little Inagua, Bahamas Islands	388	$C_{21}H_{25}ClN_2OS$	[33]
Conulothiazole B (**57**)	*S. conulosa*	Coast of Little Inagua, Bahamas Islands	402	$C_{22}H_{27}ClN_2OS$	[33]

Table S6. Terpenoids reported from genus *Smenospongia*.

Compound Name	Source	Place	Mol. Wt.	Mol. Formula	Ref.
Aureol (**58**)	*S. aurea*	Glover and Lighthouse Reefs, Belize, Caribbean Sea	314	$C_{21}H_{30}O_2$	[15,16]
	S. aurea	San Salvador Island coasts	-	-	[34]
	S. aurea	Discovery Bay, Jamaica	-	-	[18]
	Smenospongia sp.	Batanes, Philippines	-	-	[19]
	Smenospongia sp.	Green Island, Taiwan	-	-	[52]
	Smenospongia sp.	PP Island, Andaman Sea (Krabi province, Thailand)	-	-	[14]
6`-Chloroaureol (**59**)	*S. aurea*	San Salvador Island coasts	348	$C_{21}H_{29}ClO_2$	[34]
	S. aurea	Discovery Bay, Jamaica	-	-	[18]
	S. aurea	Florida Keys, USA	-	-	[17]
	Smenospongia sp.	PP Island, Andaman Sea, Krabi province, Thailand	-	-	[14]
6`-Iodoaureol (**60**)	*Smenospongia* sp.	PP Island, Andaman Sea, Krabi province, Thailand	440	$C_{21}H_{29}IO_2$	[14]
Aureol acetate (**61**)	*S. aurea*	San Salvador Island coasts	356	$C_{23}H_{32}O_3$	[34]
	S. aurea	Discovery Bay, Jamaica	-	-	[18]
	Smenospongia sp.	PP Island, Andaman Sea, Krabi province, Thailand	-	-	[14]
Chromazonarol (**62**)	*S. aurea*	Glover and Lighthouse Reefs, Belize, Caribbean Sea	314	$C_{21}H_{30}O_2$	[15]
	Smenospongia sp.	PP Island, Andaman Sea, Krabi province, Thailand	-	-	[14]
8-*Epi*-chromazonarol (**63**)	*S. aurea*	-Puerto Morelos, Mexico<br>-Glover and Lighthouse Reefs, Belize, Caribbean Sea	314	$C_{21}H_{30}O_2$	[15]
	S. aurea	San Salvador Island coasts	-	-	[34]
Smenodiol (**64**)	*Smenospongia* sp.	Therese Island, Seychelles	372	$C_{23}H_{32}O_4$	[55]
Smenospondiol (**65**)	*Smenospongia* sp.	Gulf of Aden, near Djibouti, Red Sea	372	$C_{23}H_{32}O_4$	[38
Smenohaimien D (**66**)	*S. cerebriformis*	Vinhmoc, Quangtri, Vietnam	416	$C_{24}H_{32}O_6$	[47]
Smenohaimien E (**67**)	*S. cerebriformis*	Vinhmoc, Quangtri, Vietnam	402	$C_{24}H_{34}O_5$	[47]
Polyfibrospongol A (**68**)	*S. cerebriformis*	Vinhmoc, Quangtri, Vietnam	386	$C_{24}H_{34}O_4$	[47,64]
Polyfibrospongol B (**69**)	*S. cerebriformis*	Vinhmoc, Quangtri, Vietnam	402	$C_{24}H_{34}O_5$	[47,64]
19-Hydroxy-polyfibrospongol B (**70**)	*S. cerebriformis*	Vinhmoc, Quangtri, Vietnam	418	$C_{24}H_{34}O_6$	[47,64]
Dictyoceratin C (**71**)	*S. cerebriformis*	Vinhmoc, Quangtri, Vietnam	356	$C_{23}H_{32}O_3$	[47,64]
Smenorthoquinone (**72**)	*Smenospongia* sp.	Gulf of Aden, Red Sea, near Djibouti	372	$C_{23}H_{32}O_4$	[39]
Arenarone (**73**)	*Smenospongia* sp.	Gulf of Aden, Red Sea, near Djibouti	312	$C_{21}H_{28}O_2$	[39]
Avarone (**74**)	*Smenospongia* sp.	Gulf of Aden, Red Sea, near Djibouti	312	$C_{21}H_{28}O_2$	[39]
Ilimaquinone (**75**)	*Smenospongia* sp.	Gulf of Aden, Red Sea, near Djibouti	358	$C_{22}H_{30}O_4$	[39,28]
	S. cerebriformis	-	-	-	[43]
Smenoquinone (**76**)	*Smenospongia* sp.	Gulf of Aden, Red Sea, near Djibouti	344	$C_{21}H_{28}O_4$	[39]
(+)-5-*Epi*-20-O-ethylsmenoquinone (**77**)	*S. aurea*<br>*S. cerebriformis*	Key Largo, FL, Florida key, USA	372	$C_{23}H_{32}O_4$	[46]
Smenoqualone (**78**)	*Smenospongia* sp.	Gulf of Aden, Red Sea, near Djibouti	358	$C_{22}H_{30}O_4$	[41]
Dactyloquinone C (**79**)	*S. cerebriformis*	Quang Tri, Vietnam	356	$C_{22}H_{28}O_4$	[48]

	S. cerebriformis	Vinhmoc, Quangtri, Vietnam	-	-	[60]
Dactyloquinone D (**80**)	*S. cerebriformis*	Quang Tri, Vietnam	356	$C_{22}H_{28}O_4$	[48]
	S. cerebriformis	Vinhmoc, Quangtri, Vietnam	-	-	[60]
Neodactyloquinone (**81**)	*S. cerebriformis*<br>*S. cerebriformis*	Quang Tri, Vietnam<br>Vinhmoc, Quangtri, Vietnam	356<br>-	$C_{22}H_{28}O_4$<br>-	[48]<br>[63]
Smenospongine (**82**)	*Smenospongia* sp.<br>*S. cerebriformis*	Gulf of Aden, Red Sea, near Djibouti,<br>Vinhmoc, Quangtri, Vietnam	343<br>-	$C_{21}H_{29}NO_3$<br>-	[38,39<br>[47]
Smenohaimien F (**83**)	*S. cerebriformis*	Quang Tri, Vietnam	341	$C_{21}H_{27}NO_3$	[48]
Smenospongorine (**84**)	*Smenospongia* sp.	Gulf of Aden, near Djibouti, Red Sea	399	$C_{25}H_{37}NO_3$	[39]
Smenospongiarine (**85**)	*Smenospongia* sp.	Gulf of Aden, near Djibouti, Red Sea	413	$C_{26}H_{39}NO_3$	[39]
Smenospongidine (**86**)	*Smenospongia* sp.	Gulf of Aden, near Djibouti, Red Sea	447	$C_{29}H_{37}NO_3$	[39]
(-)-Nakijinol E (**87**)	*S. aurea*<br>*S. cerebriformis*	Key Largo, FL, Florida key, USA	383	$C_{24}H_{33}NO_3$	[46]
(+)-5-*Epi*-nakijinol E (**88**)	*S. aurea*<br>*S. cerebriformis*	Key Largo, FL, Florida key, USA	383	$C_{24}H_{33}NO_3$	[46]
Nakijinone A (**89**)	*S. aurea*<br>*S. cerebriformis*	Key Largo, FL, Florida key, USA	397	$C_{25}H_{35}NO_3$	[46]
5-*Epi*-nakijinone A (**90**)	*S. aurea*<br>*S. cerebriformis*	Key Largo, FL, Florida key, USA	397	$C_{25}H_{35}NO_3$	[46]
Smenohaimien C (**91**)	*S. cerebriformis*	Vinhmoc, Quangtri, Vietnam	369	$C_{23}H_{31}NO_3$	[47]
Smenotronic acid (**92**)	*Smenospongia* sp.	Gulf of Aden, near Djibouti, Red Sea	362	$C_{21}H_{30}O_5$	[49]
Dactylospongenone A (**93**)	*S. cerebriformis*	Sea of Quangtri, Vietnam	390	$C_{23}H_{34}O_5$	[35]
Dactylospongenone B (**94**)	*S. cerebriformis*	Sea of Quangtri, Vietnam	390	$C_{23}H_{34}O_5$	[35]
Dactylospongenone C (**95**)	*S. cerebriformis*	Sea of Quangtri, Vietnam	390	$C_{23}H_{34}O_5$	[35]
Dactylospongenone D (**96**)	*S. cerebriformis*	Sea of Quangtri, Vietnam	390	$C_{23}H_{34}O_5$	[35]
(-)-Dactylospongenone E (**97**)	*S. aurea*<br>*S. cerebriformis*	Key Largo, FL, Florida key, USA	404	$C_{24}H_{36}O_5$	[46]
5-*Epi*-dactylospongenone E (**98**)	*S. aurea*<br>*S. cerebriformis*	Key Largo, FL, Florida key, USA	404	$C_{24}H_{36}O_5$	[46]
5-*Epi*-dactylospongenone F (**99**)	*S. aurea*<br>*S. cerebriformis*	Key Largo, FL, Florida key, USA	404	$C_{24}H_{36}O_5$	[46]
Smenohaimien A (**100**)	*S. cerebriformis*	Vinhmoc, Quangtri, Vietnam	332	$C_{21}H_{32}O_3$	[47]
Smenohaimien B (**101**)	*S. cerebriformis*	Vinhmoc, Quangtri, Vietnam	332	$C_{21}H_{32}O_3$	[47]
6`-Aureoxyaureol (**102**)	*Smenospongia* sp.	PP Island, Andaman Sea, Krabi province, Thailand	626	$C_{42}H_{58}O_4$	[14]
Diterpenoids					
Amijiol (**103**)	*S. cerebriformis*	Quang Tri, Vietnam	304	$C_{20}H_{32}O_2$	[48]
Isoamijiol (**104**)	*S. cerebriformis*	Quang Tri, Vietnam	304	$C_{20}H_{32}O_2$	[48]
Sesterterpenoids					
Manoalide (**105**)	*Smenospongia* sp.	Ninami-jima Island, Nichinan-oshima Island	416	$C_{25}H_{36}O_5$	[53]
seco-Manoalide (**106**)	*Smenospongia* sp.	Ninami-jima Island, Nichinan-oshima Island	416	$C_{25}H_{36}O_5$	[53]
Manoalide 25-acetate (**107**)	*Smenospongia* sp.	Ninami-jima Island, Nichinan-oshima Island	458	$C_{27}H_{38}O_6$	[53]
(4*E*,6*E*)-Dehydromanoalide (**108**)	*Smenospongia* sp.	Ninami-jima Island, Nichinan-oshima Island	398	$C_{25}H_{34}O_4$	[53]
Furospinulosin 1 (**109**)	*Smenospongia* sp.<br>*Smenospongia* sp.	Batanes, Philippines<br>PP Island, Andaman Sea, Krabi province, Thailand	354<br>-	$C_{25}H_{38}O$<br>-	[19]<br>[14]

4-Hydroxy-9-deoxoidiadione (**110**)	*Smenospongia* sp.	Shore of Gagu-Do Island, southwestern Korea	386	$C_{25}H_{38}O_3$	[35]
	Smenospongia sp.	Shore of Soheuksan Island, Korea	-	-	[54]
4-Acetoxy-9-deoxoidiadione (**111**)	*Smenospongia* sp.	Shore of Gagu-Do Island, southwestern Korea	428	$C_{27}H_{40}O_4$	[35]
7*E*,12*E*,18*R*,20*Z*-Variabilin (**112**)	*Smenospongia* sp.	Shore of Soheuksan Island, Korea	398	$C_{25}H_{34}O_4$	[54]
7E,13Z,18*R*,20Z-Felixinin (**113**)	*Smenospongia* sp.	Shore of Soheuksan Island, Korea	398	$C_{25}H_{34}O_4$	[54]
8*E*,13*Z*,18*R*,20*Z*-Strobilinin (**114**)	*Smenospongia* sp.	Shore of Soheuksan Island, Korea	398	$C_{25}H_{34}O_4$	[54]
8*Z*,13*Z*,18*R*,20*Z*-Strobilinin (**115**)	*Smenospongia* sp.	Shore of Soheuksan Island, Korea	398	$C_{25}H_{34}O_4$	[54]
12-Deacetoxy-23-acetoxyscalarin (**116**)	*Smenospongia* sp.	Shore of Gagu-Do Island, southwestern Korea	444	$C_{27}H_{40}O_5$	[35]
	Smenospongia sp.	Shore of Soheuksan Island, Korea	-	-	[54]
	Smenospongia sp.	Gageo Island, South Korea	-	-	[53]
12-Deacetoxy-23-acetoxy-19-O-acetylscalarin (**117**)	*Smenospongia* sp.	Shore of Gagu-Do Island, southwestern Korea	486	$C_{29}H_{42}O_6$	[35]
	Smenospongia sp.	Shore of Soheuksan Island, Korea	-	-	[54]
12-Deacetoxy-19-O-acetyl-23-hydroxyscalarin (**118**)	*Smenospongia* sp.	Shore of Soheuksan Island, Korea	444	$C_{27}H_{40}O_5$	[54]
12-Deacetoxy-23-hydroxy-19-O-methylscalarin (**119**)	*Smenospongia* sp.	Shore of Soheuksan Island, Korea	402	$C_{26}H_{42}O_3$	[54]
12-Deacetoxy-23-hydroxyheteronemin (**120**)	*Smenospongia* sp.	Shore of Gagu-Do Island, southwestern Korea	488	$C_{29}H_{44}O_6$	[35]
	Smenospongia sp.	Gageo Island, South Korea	-	-	[53]
12-Deacetoxy-23-acetoxyheteronemin (**121**)	*Smenospongia* sp.	Shore of Soheuksan Island, Korea	530	$C_{31}H_{46}O_7$	[54]
	Smenospongia sp.	Gageo Island, South Korea	-	-	[53]
12-Deacetoxy-19-O-acetyl-16-deacetoxyheteronemin (**122**)	*Smenospongia* sp.	Shore of Soheuksan Island, Korea	430	$C_{27}H_{42}O_4$	[54]
12-Deacetoxy-23-aldehydeheteronemin (**123**)	*Smenospongia* sp.	Shore of Soheuksan Island, Korea	500	$C_{30}H_{44}O_6$	[54]
12-Deacetoxy-23-deacetoxyscalarin (**124**)	*Smenospongia* sp.	Gageo Island, South Korea	402	$C_{25}H_{38}O_4$	[53]
12-Deacetoxy-23-hydroxyscalarafuran (**125**)	*Smenospongia* sp.	Shore of Soheuksan Island, Korea	370	$C_{25}H_{38}O_2$	[54]
12-Deacetoxy-23-acetoxyscalarafuran (**126**)	*Smenospongia* sp.	Shore of Soheuksan Island, Korea	412	$C_{27}H_{40}O_3$	[54]
18*S*-12-deacetoxy-23-acetoxy-20-carboxyscaladial (**127**)	*Smenospongia* sp.	Shore of Soheuksan Island, Korea	444	$C_{27}H_{40}O_5$	[54]
18*S*-12-deacetoxy-23-acetoxy-20-methoxyscaladial (**128**)	*Smenospongia* sp.	Shore of Soheuksan Island, Korea	458	$C_{28}H_{42}O_5$	[54]
18*R*-12-deacetoxy-23-acetoxy-20-methoxyscaladial (**129**)	*Smenospongia* sp.	Shore of Soheuksan Island, Korea	458	$C_{28}H_{42}O_5$	[54]

Table S7. Chromene derivatives reported from genus *Smenospongia*.

Compound Name	Source	Place	Mol. Wt.	Mol. Formula	Ref.
Smenochromene A (**130**)	*Smenospongia* sp.	Therese Island, Seychelles	338	$C_{22}H_{26}O_3$	[55]
Smenochromene B (**131**)	*Smenospongia* sp.	Therese Island, Seychelles	338	$C_{22}H_{26}O_3$	[55]
Smenochromene C (**132**)	*Smenospongia* sp.	Therese Island, Seychelles	340	$C_{22}H_{28}O_3$	[55]
Smenochromene D (**133**)	*Smenospongia* sp.	Therese Island, Seychelles	340	$C_{22}H_{28}O_3$	[55]

Table S8. γ- Pyrone, phenyl alkenes, and naphthoquinones derivatives reported from genus *Smenospongia*.

Compound Name	Source	Place	Mol. Wt.	Mol. Formula	Ref.
γ-Pyrone derivatives					
Smenopyrone (**134**)	*S. aurea*	coast of Great Inagua, Bahamas Islands	418	$C_{25}H_{38}O_5$	[57]
Phenyl alkene derivatives					
(*E*)-10-benzyl-5,7-dimethylun-deca-1,5,10-trien-4-ol (**135**)	*S. aurea* *S. cerebriformis*	Florida Keys, USA	284	$C_{20}H_{28}O$	[60]
Phenolics					
(*E*)-2,4-Dimethoxy-6-(3-methyl-5-(1,2,6-trimethylcyclohex -2-en-1-yl)pent-2-en-1-yl)phenol (**136**)	*S. echina*	Puerto Morelos, Mexico	358	$C_{23}H_{34}O_3$	[15]
Naphthoquinones					
Smenocerone A (**137**)	*S. cerebriformis*	Sea of Quangtri, Vietnam	278	$C_{14}H_{14}O_6$	[51]
Smenocerone B (**138**)	*S. cerebriformis*	Sea of Quangtri, Vietnam	308	$C_{15}H_{16}O_7$	[51]

Table S9. Fatty acids, sterols, and phthalates reported from genus *Smenospongia*.

Compound Name	Source	Place	Mol. Wt.	Mol. Formula	Ref.
Fatty Acids					
2-Hydroxy-17-methyloctade-canoic acid (**139**)	*S. aurea*	Shelf edge of La Pargu-era, Puerto Rica	314	$C_{19}H_{38}O_3$	[61]
2-Hydroxy-21-methyldocosa-noic acid (**140**)	*S. aurea*	Shelf edge of La Pargu-era, Puerto Rica	370	$C_{23}H_{46}O_3$	[61]
2-Hydroxy-22-methyltricosa-noic acid (**141**)	*S. aurea*	Shelf edge of La Pargu-era, Puerto Rica	384	$C_{24}H_{48}O_3$	[61]
2-Hydroxy-22-methyltetraco-sanoic acid (**142**)	*S. aurea*	Shelf edge of La Pargu-era, Puerto Rica	398	$C_{25}H_{50}O_3$	[61]
2-Hydroxy-24-methylpentaco-sanoic acid (**143**)	*S. aurea*	Shelf edge of La Pargu-era, Puerto Rica	412	$C_{26}H_{52}O_3$	[61]
2-Hydroxy-23-methylpentaco-sanoic acid (**144**)	*S. aurea*	Shelf edge of La Pargu-era, Puerto Rica	412	$C_{26}H_{52}O_3$	[61]
Linoleic acid (**145**)	*Smenospongia* sp.	El-Gouna, Hurghada-coasts, Red Sea, Egypt	280	$C_{18}H_{32}O_2$	[62]
Sterols					
β-Sitosterol (**146**)	*Smenospongia* sp.	El-Gouna, Hurghada-coasts, Red Sea, Egypt	414	$C_{29}H_{50}O$	[62]
Cholesterol (**147**)	*Smenospongia* sp.	El-Gouna, Hurghada-coasts, Red Sea, Egypt	416	$C_{29}H_{52}O$	[62]
Ergosterol (**148**)	*Smenospongia* sp.	PP Island, Andaman Sea, Krabi province, Thailand	396	$C_{28}H_{44}O$	[14]

24-Methylcholesta-5,22-dien-3β-ol (**149**)	*S. aurea*	Shelf edge of La Pargu-era, Puerto Rica	398	$C_{28}H_{46}O$	[61]
24-Methyl-cholest-5-en-3β-ol (**150**)	*S. aurea*	Shelf edge of La Pargu-era, Puerto Rica	400	$C_{28}H_{48}O$	[61]
24-Ethylcholesta-5,22-dien-3β-ol (**151**)	*S. aurea*	Shelf edge of La Pargu-era, Puerto Rica	412	$C_{29}H_{48}O$	[61]
24-Ethylcholest-5-en-3β-ol (**152**)	*S. aurea*	Shelf edge of La Pargu-era, Puerto Rica	414	$C_{29}H_{50}O$	[61]
Phthalates					
Di-isobutyl phthalate (**153**)	*Smenospongia* sp.	El-Gouna, Hurghada-coasts, Red Sea, Egypt	278	$C_{16}H_{22}O_4$	[62]
Di-*n*-butyl phthalate (**154**)	*Smenospongia* sp.	El-Gouna, Hurghada-coasts, Red Sea, Egypt	278	$C_{16}H_{22}O_4$	[62]

References

1. Ibrahim, S.R.; Mohamed, G.A. Marine pyridoacridine alkaloids: Biosynthesis and biological activities. *Chem. Biodiv.* **2016**, *13*, 37–47.
2. Ibrahim, S.R.; Fadil, S.A.; Fadil, H.A.; Hareeri, R.H.; Alolayan, S.O.; Abdallah, H.M.; Mohamed, G.A. *Dactylospongia elegans*—A promising drug source: Metabolites, bioactivities, biosynthesis, synthesis, and structural-activity relationship. *Mar. Drugs* **2022**, *20*, 221.
3. Esposito, R.; Federico, S.; Bertolino, M.; Zupo, V.; Costantini, M. Marine Demospongiae: A challenging treasure of bioactive compounds. *Mar. Drugs* **2022**, *20*, 244.
4. Perdicaris, S.; Vlachogianni, T.; Valavanidis, A. Bioactive natural substances from marine sponges: New developments and prospects for future pharmaceuticals. *Nat. Prod. Chem. Res.* **2013**, *1*, 1–8.
5. Pawlik, J.R.; McMurray, S.E. The emerging ecological and biogeochemical importance of sponges on coral reefs. *Ann. Rev. Mar. Sci.* **2020**, *12*, 315–337.
6. Maslin, M.; Gaertner-Mazouni, N.; Debitus, C.; Joy, N.; Ho, R. Marine sponge aquaculture towards drug development: An ongoing history of technical, ecological, chemical considerations and challenges. *Aquacult. Rep*. **2021**, *21*, 100813.
7. Aguila-Ramírez, R.N.; Hernández-Guerrero, C.J.; González-Acosta, B.; Id-Daoud, G.; Hewitt, S.; Pope, J.; Hellio, C. Antifouling activity of symbiotic bacteria from sponge *Aplysina gerardogreeni*. *Int. Biodeterior. Biodegrad.* **2014**, *90*, 64–70.
8. Ibrahim, S.R.; Edrada-Ebel, R.; Mohamed, G.A.; Youssef, D.T.; Wray, V.; Proksch, P. Callyaerin G, a new cytotoxic cyclic peptide from the marine sponge *Callyspongia aerizusa*. *Arkivoc* **2008**, *12*, 164–171.
9. Ibrahim, S.R.; Min, C.C.; Teuscher, F.; Ebel, R.; Kakoschke, C.; Lin, W.; Wray, V.; Edrada-Ebel, R.; Proksch, P. Callyaerins A–F and H, new cytotoxic cyclic peptides from the indonesian marine sponge *Callyspongia aerizusa*. *Bioorg. Med. Chem*. **2010**, *18*, 4947–4956.
10. Ibrahim, S.R.; Mohamed, G.A.; Zayed, M.F.; Sayed, H.M. Ingenines A and B. Two new alkaloids from the indonesian sponge *Acanthostrongylophora ingens*. *Drug Res.* **2015**, *65*, 361–365.
11. Ibrahim, S.R.; Mohamed, G.A. Ingenines C and D, new cytotoxic pyrimidine-β-carboline alkaloids from the indonesian sponge *Acanthostrongylophora ingens*. *Phytochem. Lett*. **2016**, *18*, 168–171.
12. Ibrahim, S.R.; Mohamed, G.A. Pyridoacridine alkaloids from deep-water marine organisms: Structural elucidation. *Bull. Fac. Pharm. Cairo Univ.* **2016**, *54*, 107–135.
13. Sim, C.J.; Lee, K.J.; Kim, Y.A. twelve new species of two genera *Smenospongia* and *Cacospongia* (Demospongia: Dictyoceratida: Thorectidae) from Korea. *J. Spec. Res.* **2016**, *5*, 31–48.
14. McKay, M.J.; Carroll, A.R.; Quinn, R.J.; Hooper, J.N. 1,2-Bis (1*H*-indol-3-Yl) ethane-1,2-dione, an indole alkaloid from the marine sponge *Smenospongia* sp. *J. Nat. Prod*. **2002**, *65*, 595–597.

15. Hang, D.T.T.; Nhiem, N.X.; Tai, B.H.; Anh, H.L.T.; Yen, P.H.; Van Dau, N.; Van Minh, C.; Van Kiem, P. Merosesquiterpenes from marine sponge *Smenospongia Cerebriformis*. *Vietnam J. Chem.* **2017**, *55*, 153.
16. Hu, J.; Schetz, J.A.; Kelly, M.; Peng, J.; Ang, K.K.; Flotow, H.; Leong, C.Y.; Ng, S.B.; Buss, A.D.; Wilkins, S.P. New antiinfective and human 5-HT2 receptor binding natural and semisynthetic compounds from the Jamaican sponge *Smenospongia aurea*. *J. Nat. Prod.* **2002**, *65*, 476–480.
17. Prawat, H.; Mahidol, C.; Kaweetripob, W.; Wittayalai, S.; Ruchirawat, S. Iodo–sesquiterpene hydroquinone and brominated indole alkaloids from the Thai sponge *Smenospongia* sp. *Tetrahedron* **2012**, *68*, 6881–6886.
18. Djura, P.; Stierle, D.B.; Sullivan, B.; Faulkner, D.J.; Arnold, E.V.; Clardy, J. Some metabolites of the marine sponges *Smenospongia aurea* and *Smenospongia* (Ident. *Polyfibrospongia*) *echina*. *J. Org. Chem.* **1980**, *45*, 1435–1441.
19. Tymiak, A.A.; Rinehart Jr, K.L.; Bakus, G.J. Constituents of morphologically similar sponges: *Aplysina* and *Smenospongia* species. *Tetrahedron* **1985**, *41*, 1039–1047.
20. Tasdemir, D.; Bugni, T.S.; Mangalindan, G.C.; Concepción, G.P.; Harper, M.K.; Ireland, C.M. Cytotoxic bromoindole derivatives and terpenes from the Philippine marine sponge *Smenospongia* sp. *Z. Naturforsch. C* **2002**, *57*, 914–922.
21. Teta, R.; Della Sala, G.; Esposito, G.; Via, C.W.; Mazzoccoli, C.; Piccoli, C.; Bertin, M.J.; Costantino, V.; Mangoni, A. A joint molecular networking study of a *Smenospongia* sponge and a cyanobacterial bloom revealed new antiproliferative chlorinated polyketides. *Org. Chem. Front.* **2019**, *6*, 1762–1774.
22. Aiello, A.; Fattorusso, E.; Menna, M. A new antibiotic chloro-sesquiterpene from the Caribbean sponge *Smenospongia aurea*. *Z. Naturforsch. B* **1993**, *48*, 209–212.
23. Esposito, G.; Della Sala, G.; Teta, R.; Caso, A.; Bourguet-Kondracki, M.; Pawlik, J.R.; Mangoni, A.; Costantino, V. chlorinated thiazole-containing polyketide-peptides from the Caribbean sponge *Smenospongia conulosa*: Structure elucidation on microgram scale. *Eur. J. Org. Chem.* **2016**, *2016*, 2871–2875.
24. Shen, Y.; Liaw, C.; Ho, J.; Khalil, A.T.; Kuo, Y. Isolation of aureol from *Smenospongia* sp. and cytotoxic activity of some aureol derivatives. *Nat. Prod. Res.* **2006**, *20*, 578–585.
25. Venkateswarlu, Y.; Faulkner, D.J.; Steiner, J.L.R.; Corcoran, E.; Clardy, J. Smenochromenes, unusual macrocyclic sesquiterpene hydroquinone derivatives from a Seychelles sponge of the genus *Smenospongia*. *J. Org. Chem.* **1991**, *56*, 6271–6274.
26. Kondracki, M.; Guyot, M. Biologically active quinone and hydroquinone sesquiterpenoids from the sponge *Smenospongia* sp. *Tetrahedron* **1989**, *45*, 1995–2004.
27. Van Kiem, P.; Hang, D.T.; Nhiem, N.X.; Tai, B.H.; Anh, H.L.T.; Van Cuong, P.; Quang, T.H.; Van Minh, C.; Van Dau, N.; Kim, Y. Sesquiterpene derivatives from marine sponge *Smenospongia cerebriformis* and their anti-inflammatory activity. *Bioorg. Med. Chem. Lett.* **2017**, *27*, 1525–1529.
28. Hang, D.T.T.; Nhiem, N.X.; Tai, B.H.; Anh, H.L.T.; Yen, P.H.; Van Dau, N.; Van Minh, C.; Van Kiem, P. Sesquiterpene phenols from marine sponge *Smenospongia cerebriformis*. *Vietnam J. Chem.* **2017**, *55*, 148.
29. Bourguet-Kondracki, M.; Guyot, M. A new sesquiterpene tetronic acid derivative from the marine sponge *Smenospongia* sp. *Tetrahedron Lett.* **1999**, *40*, 3149–3150.
30. Kwak, C.; Jin, L.; Han, J.H.; Han, C.W.; Kim, E.; Cho, M.; Chung, T.; Bae, S.; Jang, S.B.; Ha, K. Ilimaquinone induces the apoptotic cell death of cancer cells by reducing pyruvate dehydrogenase kinase 1 activity. *Int. J. Mol. Sci.* **2020**, *21*, 6021.
31. Hwang, I.H.; Oh, J.; Zhou, W.; Park, S.; Kim, J.; Chittiboyina, A.G.; Ferreira, D.; Song, G.Y.; Oh, S.; Na, M. Cytotoxic activity of rearranged drimane meroterpenoids against colon cancer cells via down-regulation of β-catenin expression. *J. Nat. Prod.* **2015**, *78*, 453–461.
32. Bourguet-Kondracki, M.; Martin, M.; Guyot, M. Smenoqualone a novel sesquiterpenoid from the marine sponge *Smenospongia* sp. *Tetrahedron Lett.* **1992**, *33*, 8079–8080.
33. Huyen, L.T.; Hang, D.T.; Nhiem, N.X.; Tai, B.H.; Anh, H.L.T.; Quang, T.H.; Yen, P.H.; Van Minh, C.; Van Dau, N.; Van Kiem, P. Sesquiterpene quinones and diterpenes from *Smenospongia cerebriformis* and their cytotoxic activity. *Nat. Prod. Commun.* **2017**, *12*, 1934578X1701200402.
34. Kochanowska, A.J.; Rao, K.V.; Childress, S.; El-Alfy, A.; Matsumoto, R.R.; Kelly, M.; Stewart, G.S.; Sufka, K.J.; Hamann, M.T. Secondary metabolites from three Florida sponges with antidepressant activity. *J. Nat. Prod.* **2008**, *71*, 186–189.
35. Kobayashi, J.; Murayama, T.; Ishibashi, M.; Kosuge, S.; Takamatsu, M.; Ohizumi, Y.; Kobayashi, H.; Ohta, T.; Nozoe, S.; Takuma, S. Hyrtiosins A and B, new indole alkaloids from the okinawan marine sponge *Hyrtios erecta*. *Tetrahedron* **1990**, *46*, 7699–7702.
36. Swain, S.P.; Mohanty, S. Imidazolidinones and imidazolidine-2,4-diones as antiviral agents. *ChemMedChem* **2019**, *14*, 291–302.
37. Dai, J.; Jiménez, J.I.; Kelly, M.; Barnes, S.; Lorenzo, P.; Williams, P. Dictazolines A and B, bisspiroimidazolidinones from the marine sponge *Smenospongia cerebriformis*. *J. Nat. Prod.* **2008**, *71*, 1287–1290.
38. Hwang, I.H.; Oh, J.; Kochanowska-Karamyan, A.; Doerksen, R.J.; Na, M.; Hamann, M.T. A novel natural phenyl alkene with cytotoxic activity. *Tetrahedron Lett.* **2013**, *54*, 3872–3876.
39. Le, T.H.; Hang, D.T.T.; Nhiem, N.X.; Yen, P.H.; Anh, H.L.T.; Quang, T.H.; Tai, B.H.; Van Dau, N.; Van Kiem, P. Naphtoquinones and sesquiterpene cyclopentenones from the sponge *Smenospongia cerebriformis* with their cytotoxic activity. *Chem. Pharm. Bull.* **2017**, *65*, 589–592.

40. Caso, A.; Mangoni, A.; Piccialli, G.; Costantino, V.; Piccialli, V. Studies toward the synthesis of smenamide a, an antiproliferative metabolite from *smenospongia aurea*: Total synthesis of ent-smenamide A and 16-epi-smenamide A. *Acs Omega* **2017**, *2*, 1477–1488.
41. Esposito, G.; Teta, R.; Della Sala, G.; Pawlik, J.R.; Mangoni, A.; Costantino, V. Isolation of smenopyrone, a bis-γ-pyrone polypropionate from the Caribbean sponge *Smenospongia aurea*. *Mar. Drugs* **2018**, *16*, 285.
42. Caso, A.; Laurenzana, I.; Lamorte, D.; Trino, S.; Esposito, G.; Piccialli, V.; Costantino, V. Smenamide A analogues. synthesis and biological activity on multiple myeloma cells. *Mar. Drugs* **2018**, *16*, 206.
43. Via, C.W.; Glukhov, E.; Costa, S.; Zimba, P.V.; Moeller, P.D.; Gerwick, W.H.; Bertin, M.J. The metabolome of a cyanobacterial bloom visualized by MS/MS-based molecular networking reveals new neurotoxic smenamide analogs (C. D. and E). *Front. Chem.* **2018**, *6*, 316.
44. Song, J.; Jeong, W.; Wang, N.; Lee, H.; Sim, C.J.; Oh, K.; Shin, J. Scalarane sesterterpenes from the sponge *Smenospongia* sp. *J. Nat. Prod.* **2008**, *71*, 1866–1871.
45. Wang, J.; Li, H.; Wang, M.; Wang, J.; Wu, Y. A six-step synthetic approach to marine natural product (-)-aureol. *Tetrahedron Lett.* **2018**, *59*, 945–948.
46. Hwang, B.; Rho, J. Scalaran-type sesterterpenes from a marine sponge *Smenospongia* species showing the AMPK sctivation. *J. Korean Magn. Reson. Soc.* **2012**, *16*, 1–10.
47. Teta, R.; Irollo, E.; Della Sala, G.; Pirozzi, G.; Mangoni, A.; Costantino, V. Smenamides A and B, chlorinated peptide/polyketide hybrids containing a dolapyrrolidinone unit from the Caribbean sponge *Smenospongia aurea*. evaluation of their role as leads in antitumor drug research. *Mar. Drugs* **2013**, *11*, 4451–4463.
48. Esposito, G.; Teta, R.; Miceli, R.; Ceccarelli, L.S.; Della Sala, G.; Camerlingo, R.; Irollo, E.; Mangoni, A.; Pirozzi, G.; Costantino, V. Isolation and assessment of the in vitro anti-tumor activity of smenothiazole A and B, chlorinated thiazole-containing peptide/polyketides from the Caribbean sponge, *Smenospongia aurea*. *Mar. Drugs* **2015**, *13*, 444–459.
49. Rho, J.; Lee, H.; Shin, H.J.; Ahn, J.; Kim, J.; Sim, C.J.; Shin, J. New sesterterpenes from the sponge *Smenospongia* sp. *J. Nat. Prod.* **2004**, *67*, 1748–1751.
50. Wright, A.E.; Rueth, S.A.; Cross, S.S. An antiviral sesquiterpene hydroquinone from the marine sponge *Strongylophora hartmani*. *J. Nat. Prod.* **1991**, *54*, 1108–1111.
51. Carballelra, N.M.; Emiliano, A.; Rodriguez, J.; Reyes, E.D. Isolation and characterization of novel 2-hydroxy fatty acids from the phospholipids of the sponge *Smenospongia aurea*. *Lipids* **1992**, *27*, 681–685.
52. Shaaban, M.; Abd-Alla, H.I.; Hassan, A.Z.; Aly, H.F.; Ghani, M.A. Chemical characterization, antioxidant and inhibitory effects of some marine sponges against carbohydrate metabolizing enzymes. *Org. Med. Chem. Lett.* **2012**, *2*, 30.
53. Bialonska, D.; Zjawiony, J.K. Aplysinopsins-marine indole alkaloids: Chemistry, bioactivity and ecological significance. *Mar. Drugs* **2009**, *7*, 166–183.
54. Caso, A.; Esposito, G.; Della Sala, G.; Pawlik, J.R.; Teta, R.; Mangoni, A.; Costantino, V. Fast detection of two smenamide family members using molecular networking. *Mar. Drugs* **2019**, *17*, 618.
55. Kondracki, M.; Guyot, M. Smenospongine: A cytotoxic and antimicrobial aminoquinone isolated from *Smenospongia* sp. *Tetrahedron Lett.* **1987**, *28*, 5815–5818.
56. Do, M.T.; Na, M.; Kim, H.G.; Khanal, T.; Choi, J.H.; Jin, S.W.; Oh, S.H.; Hwang, I.H.; Chung, Y.C.; Kim, H.S. Ilimaquinone induces death receptor expression and sensitizes human colon cancer cells to trail-induced apoptosis through activation of ROS-ERK/p38 MAPK–CHOP signaling pathways. *Food Chem. Toxicol.* **2014**, *71*, 51–59.
57. Atas, E.; Oberhuber, M.; Kenner, L. The implications of PDK1–4 on tumor energy metabolism, aggressiveness and therapy resistance. *Front. Oncol.* **2020**, *10*, 583217.
58. Son, Y.; Lim, D.; Park, S.; Song, I.; Kim, J.; Shin, S.; Jang, H.; Liu, K.; Yuseok, O.; Song, G. Ilimaquinone inhibits neovascular age-related macular degeneration through modulation of Wnt/B-catenin and p53 pathways. *Pharmacol. Res.* **2020**, *161*, 105146.
59. López, M.D.; Quiñoá, E.; Riguera, R.; Omar, S. Dactyltronic acids from the sponge *Dactylospongia elegans*. *J. Nat. Prod.* **1994**, *57*, 992–996.
60. Rosa, C.P.; Kienzler, M.A.; Olson, B.S.; Liang, G.; Trauner, D. Total synthesis of smenochromene B through ring contraction. *Tetrahedron* **2007**, *63*, 6529–6534.
61. Lin, Z.; Torres, J.P.; Ammon, M.A.; Marett, L.; Teichert, R.W.; Reilly, C.A.; Kwan, J.C.; Hughen, R.W.; Flores, M.; Tianero, M.D. A bacterial source for mollusk pyrone polyketides. *Chem. Biol.* **2013**, *20*, 73–81.
62. Zhou, Z.; Li, X.; Yao, L.; Li, J.; Gavagnin, M.; Guo, Y. Marine bis-Γ-pyrone polypropionates of onchidione family and their effects on the XBP1 gene expression. *Bioorg. Med. Chem. Lett.* **2018**, *28*, 1093–1096.
63. Segraves, N.L.; Crews, P. Investigation of brominated tryptophan alkaloids from two Thorectidae sponges: *Thorectandra* and *Smenospongia*. *J. Nat. Prod.* **2005**, *68*, 1484–1488.
64. Tsukamoto, S.; Kato, H.; Hirota, H.; Fusetani, N. Antifouling terpenes and steroids against barnacle larvae from marine sponges. *Biofouling* **1997**, *11*, 283–291.
